# Water Use Characteristics of Weeds: A Global Review, Best Practices, and Future Directions

**DOI:** 10.3389/fpls.2021.794090

**Published:** 2022-01-07

**Authors:** Mandeep Singh, Meetpal Singh Kukal, Suat Irmak, Amit J. Jhala

**Affiliations:** ^1^Department of Agronomy and Horticulture, University of Nebraska-Lincoln, Lincoln, NE, United States; ^2^Department of Agricultural and Biological Engineering, The Pennsylvania State University, State College, PA, United States

**Keywords:** water use, crop-weed competition, evapotranspiration, transpiration, weeds, crop-weed interactions

## Abstract

Weeds usually penalize crop yields by competing for resources, such as water, light, nutrients, and space. Most of the studies on the crop-weed competition domain are limited to assessing crop-yield losses due to weed pressure and other crop-weed interactions, overlooking the significant uptake of soil-water by weeds that exacerbates global water constraints and threatens the productivity and profitability. The objective of this review was to synthesize globally available quantitative data on weed water use (WU) sourced from 23 peer-reviewed publications (filtered from 233 publications *via* a multi-step protocol of inclusion criteria) with experimental investigations across space (3 continents), time (1927–2018), weed species (27 broadleaf and 7 grasses) and characteristics, cropping systems (5), soil types (ranging from coarse-textured sand to fine-textured clay soils), determination techniques, experimental factors (environment, management, resource availability, and competition), and aridity regimes (ranging from semi-arid to humid climate). Distributions of weed WU data reported *via eight* different metrics were assessed for variability and mean WU. A lack of the best experimental and reporting practices in weed WU research was identified that undermined the robustness, transferability, and application of the WU data. Mandatory protocols and the best practices typically followed in the agricultural water management research were described and recommended for weed scientists to avoid pitfalls in quantifying and presenting weed WU. A model of mixed plant community evapotranspiration (ET) was adapted to model weed-crop-soil system evaporation and transpiration in a crop canopy infested with multiple (*n*) weed species. Finally, potential cross-disciplinary questions across the domains of crop science, weed science, agricultural water management, irrigation science and engineering, and environmental changes were proposed to direct and prioritize future research efforts in the crop-weed-water arena.

## Introduction

Weeds represent some of the most vital biotic limitation to global crop production, with the greatest potential for crop yield penalties. Compared to animal pests (18%) and pathogens (16%), weeds can cause twice as severe (34%) of a yield penalty (Oerke, 2006). The Weed Loss Committee of the Weed Science Society of America estimates that uncontrolled weeds can reduce crop yields up to 50% in maize (*Zea mays* L.) (Soltani et al., 2016) and 52% in soybean [*Glycine max* (L.) Merrill] (Soltani et al., 2017). In India, weeds cause 36% of yield losses in peanut (*Arachis hypogaea* L.), 31% in soybean, 25% in maize and sorghum [*Sorghum bicolor* (L.) Moench], and 19% in wheat (*Triticum aestivum* L.) (Gharde et al., 2018). These yield penalties are translated into substantial financial losses, which are estimated at $26.7 and $17.2 billion for maize and soybean alone, respectively, in the US and Canada, $11 billion for 10 major crops in India, and $2.6 billion for cereals, sorghum, canola, pulses, and fallow in Australia (Llewellyn et al., 2016; Soltani et al., 2016, 2017; Gharde et al., 2018). Chauhan (2020) estimated a loss of ~200 million metric tons of grain globally due to weed pressure and competition within cropping systems, translating it into an economic loss of more than $100 billion annually (Appleby et al., 2001).

Weeds present competition for primary resources, such as water, light, nutrients, and space that limit agricultural production. Crop-weed competition and its implications have largely been expressed through the main crop rather than the weeds; hence, the vast majority of these implications are expressed as reduced crop productivity or yield (Zimdahl, 2007). However, farm-level profitability is not solely driven by crop yield, but by a net balance of costs and benefits. In ecosystems where water is a limiting resource, net profitability is strongly driven by water-related costs under both rainfed and irrigated conditions. Uncontrolled weed growth can add direct irrigation costs of more than $50 ha^−1^, while even weed densities below economic thresholds can add ~$20 ha^−1^ in production costs depending upon the cropping system and water cost (Norris, 1996). Climate change, rapidly growing populations, and environmental degradation have increased pressure over limited water resources (WRI., 2018). In this regard, increased pressure from water competition for weeds in a field will exacerbate the conditions of turgor loss, stomata closure, decreased photosynthesis, and transpiration, halting cell enlargement and metabolic processes and causing suppressed plant growth and development and eventually reducing the yield performance (Kramer and Boyer, 1995). Thus, to comprehensively assess their impacts on agroecosystems, weeds should be considered a significant source of water consumption in addition to penalizing crop productivity.

Water use (WU) of weeds is one of the most critical pieces of information underlying this assessment and the subsequent management of weeds. Owing to their superior ability for soil water exploration (Stuart et al., 1984), greater effective root zone and soil volume per plant, rapid development of extensive root systems, greater resource affinity, and higher tolerance to climatic variation than most crops (Zimdahl, 2018), weeds often demand more water than many crops. WU of weeds can vary substantially depending upon the species (Lopes et al., 2004; Pivec and Brant, 2009), photosynthetic pathway (Norris, 1996), plant architecture (Berger et al., 2015), root length and distribution (Zollinger and Kells, 1991; Berger et al., 2015; Vaughn et al., 2016), environmental factor, and management system (Massinga et al., 2003; Lopes et al., 2004; Berger et al., 2015), among other factors. These factors are not only specific to weeds, but to all the functional types of plants, including agricultural crops. Thus, crop WU and weed WU processes in crop-weed systems are intertwined in production-scale fields, owing to weed-crop competition and overlapping drivers.

Measurement, estimation, and effective communication of crop WU or crop evapotranspiration (ET) follow a standard practice, as recommended by task committees, manuals of practice, and scientific reports (Doorenbos and Kassam, 1979; Walter et al., 2000; Jensen and Allen, 2016; Pereira et al., 2021; Rallo et al., 2021). However, all the limited studies that have been done to measure and estimate WU characteristics of weeds do not seem to adhere to such a standardized protocol. This might be a consequence of disconnect among researchers and expertise in weed science, agricultural water management, soil and water resource engineering, and irrigation science and engineering. Thus, this disconnect, and the lack of inter-disciplinary collaboration has hindered the standardization of WU concepts, metrics, approaches, and applications originally developed for cash crops that are to be adopted and translated for weeds. This hindrance has consequently prevented representative, accurate, robust, and transferrable WU of weed species under different climate, soil, and management conditions.

The best practices in the measurement, estimation, and reporting of weed WU will not only strengthen the confidence and representativeness of information, but it will also allow the users to interpret, compare, contrast, and utilize WU data more effectively. This is an especially desirable quality for weed WU data due to the higher potential for their incorporation in building and parametrization of weed-oriented modules in crop models (Whish et al., 2015; Chauhan, 2020; Singh et al., 2020; Colbach et al., 2021). Currently, modeling and simulation of weed interference with cropping system performance is an emerging area that would greatly benefit from the measured data on weed WU.

This review is initially set to gather and publish a compendium of WU estimates of all possible weeds either individually or in the cropping systems across the global literature. This critical information on the crop-weed competition research area has not previously been systematically identified, collected, synthesized, and analyzed, making it important to identify evidence-based practical research voids, flaws, and knowledge gaps, and provide informed future insights, requirements, and directions for future research. Thus, the objectives of this comprehensive review were to: (i) conduct the first global review of weed WU research to compile and synthesize quantitative estimates, methodologies, and drivers of WU in weed species, (ii) discuss the merits, limitations, past and current knowledge gaps, and future directions in the relevant global research; and (iii) present and translate standard concepts, approaches, and metrics of agricultural crop WU research and practice on weed management.

## Materials and Methods

### Literature Search and Selection Criteria

**Figure 1:**
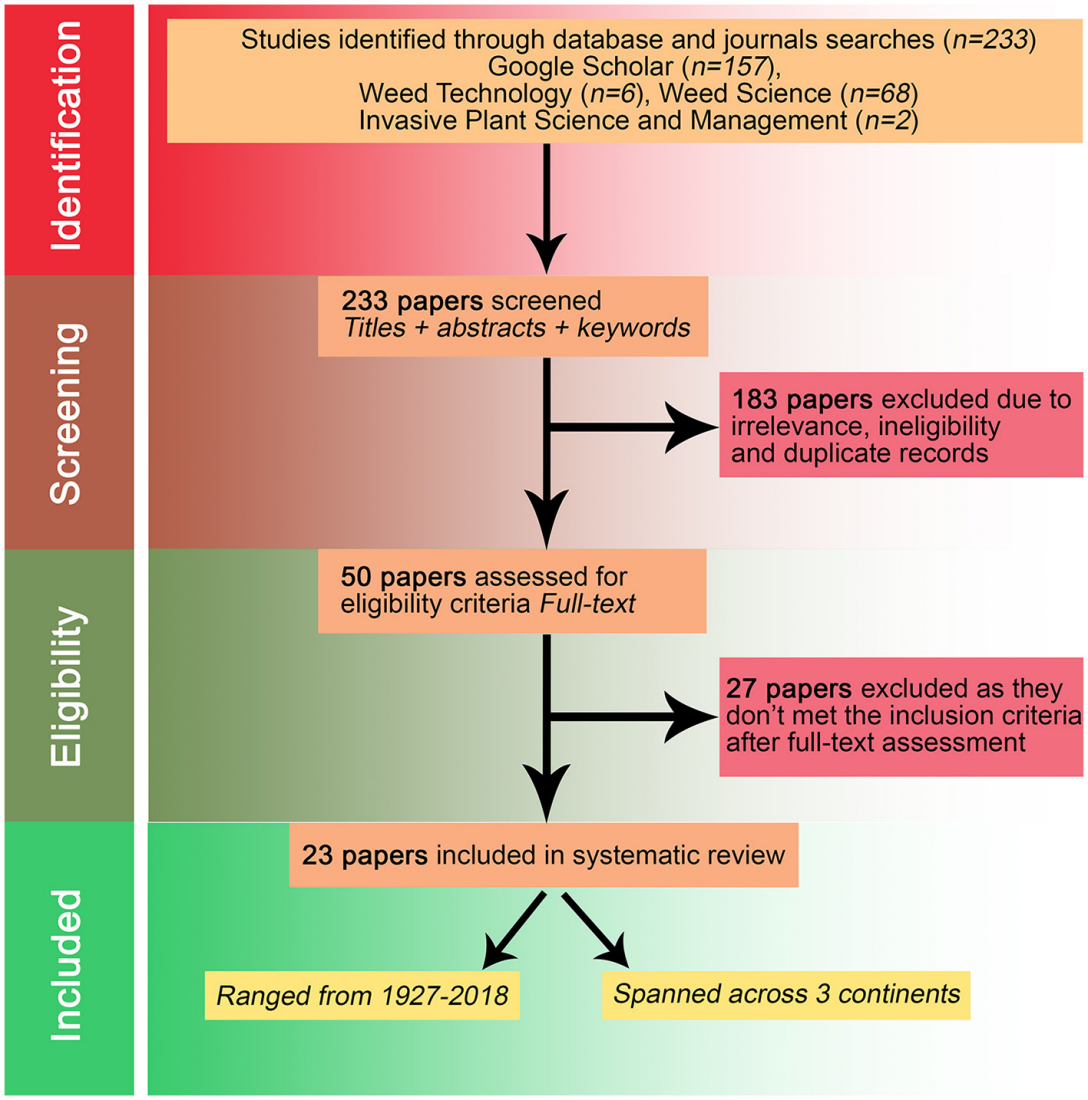
Preferred Reporting Items for Systematic Reviews and Meta-Analyses (PRISMA) diagram outlining the selection procedure for papers reporting the findings of experiments conducted on the water use (WU) of the weeds.

We used multiple-step systematic review protocol to search, select, and compile relevant publications for the review as described in the flow-diagram of Preferred Reporting Items for Systematic Reviews and Meta-Analyses (PRISMA; Page et al., 2021) presented in Figure 1. The primary literature was searched using the Google Scholar database with the following search terms: (“Weed”) AND (“Evapotranspiration” OR “Water Use” OR “Water Loss” OR “Sap Flow” OR “Transpiration” OR “Bowen Ratio”) (Supplementary Table 1). We included the topmost 10 common and troublesome weeds among all broadleaf crops, fruits, and vegetables from the 2019 Weed Science Society of America (WSSA) National Weed Survey dataset (Wychen, 2019), and the topmost 10 common and troublesome weeds among all grass crops, pasture, and turf from the 2020 WSSA National Weed Survey dataset (Wychen, 2020), as our search terms, to broaden our search criteria. The search term, “Weed” was replaced with the common and the scientific names of each individual weed species from the survey list to target search queries for these weeds (Supplementary Table 1). Further, we also searched the term, “Transpiration” in “Weed Science,” “Weed Technology,” and “Invasive Plant Science and Management” journals to inclusively gather the literature on this topic in the major peer-reviewed Weed Science scientific journals. The search queries were made in April 2020 and no language or time of publication restrictions were applied. The primary literature search terms were targeted on the title of the publication and returned an initial collection of 233 publications. The collected articles were screened based on the following selection criteria: (i) the studies considered the species to be the weed as investigated either individually or with a crop, (ii) the studies measured/estimated one or more WU metrics of the weed, and (iii) the studies included either greenhouse or field-based research. A total of 50 publications qualifying under this search criteria or indicating the existence of such data were critically reviewed. On further screening, studies in which weed species were intended as turfgrass, pasture, or seed crops and focused on soil moisture dynamics were eliminated, narrowing the literature to 23 peer-reviewed papers published between 1927 and 2018. The papers were thoroughly screened to obtain qualitative and quantitative information, such as experimental location, weeds and crops studied, factors investigated, levels of experimental factors, environmental and soil conditions, methods, and the measured values of weed WU metrics and transpiration efficiency. The list of 23 publications with reference codes, actual references, and countries/regions, where experiments were conducted, are presented in Table 1. Additional information pertaining to each publication, including the weed species studied, cropping system, annual weather [temperature, precipitation, grass-reference evapotranspiration (ET_o_), aridity index (AI), vapor pressure deficit; VPD], and soil type are presented in Supplementary Table 2.

**Table 1:** List of research studies selected and included in this systematic review (*n* = 23 papers), with reference code, name and year of publication, and country of the research study.

**Reference code**	**Reference (Publication and Year)**	**Country**
A	Shantz and Piemeisel, 1927	United States
B	Dillman, 1931	United States
C	Chow et al., 1966	United States
D	Stutte and Weiland, 1978	United States
E	Patterson and Flint, 1982	United States
F	Stuart et al., 1984	United States
G	Munger et al., 1987	United States
H	Gealy, 1987	United States
I	Gealy, 1988	United States
J	Gealy, 1989	United States
K	Trimmer and Linscott, 1990	United States
L	Zollinger and Kells, 1991	United States
M	Gealy et al., 1991	United States
N	Holloway and Shaw, 1996	United States
O	Jones et al., 1997	United States
P	Lucero et al., 2000	France
Q	Massinga et al., 2003	United States
R	Pandey et al., 2003	India
S	Lopes et al., 2004	Germany
T	Pivec and Brant, 2009	Czech Republic
U	Berger et al., 2015	United States
V	Vaughn et al., 2016	United States
W	Prince et al., 2018	United States

### Compilation and Homogenization of WU Estimates in Literature

A large range of variation was noted in different definitions and units of WU reported in the literature. WU estimates reported in the literature were classified and reported as eight primary units/groups: (i) mass-based WU (g d^−1^ plant^−1^), (ii) volume-based WU (ml d^−1^ plant^−1^), (iii) mass-based Transpiration flux (μg cm^−2^ s^−1^), (iv) molar-based transpiration flux (mol m^−2^ s^−1^), (v) depth-based WU (cm), (vi) stomatal resistance (s cm^−1^), (vii) stomatal conductance (cm s^−1^), and (viii) WU efficiency^−1^ (ml g^−1^). Four of these eight groups (i–iv) had estimates that were reported in dissimilar units. These inconsistent units were homogenized for fair intercomparison by converting all estimates into a single common unitary system. This step ensured the consistency that is necessary to draw further inferences, such as the range and summary statistics of the WU metrics. Supplementary Table 3 presents the original (as reported in the literature) and homogenized unitary systems of these four WU reporting groups.

### Long-Term Mean Meteorological Drivers at Experimental Sites

We obtained long-term (1981-2010) means (normals) of relevant climatic variables governing the WU at all of the experimental sites. These variables included annual average air temperature (T) (°C), annual precipitation (mm), annual ET_o_, annual mean AI (unitless), and annual mean VPD (kPa). The source for these datasets was Terraclimate (Abatzoglou et al., 2018), which is a gridded data product of high-spatial resolution (1/24°, ~4-km) of climate and climatic water balance for global terrestrial surfaces. TerraClimate is based on employing climatically aided interpolation to combine high-spatial resolution to climatological normals from the WorldClim dataset, with coarser resolution time varying data from other sources. The climatological gridded surfaces of the abovementioned variables were imported into ArcMap 10.7 (ESRI, Redland, CA) for data extraction at the experimental sites globally.

## Results and Discussion

### Range of Experimental Conditions

**Figure 2:**
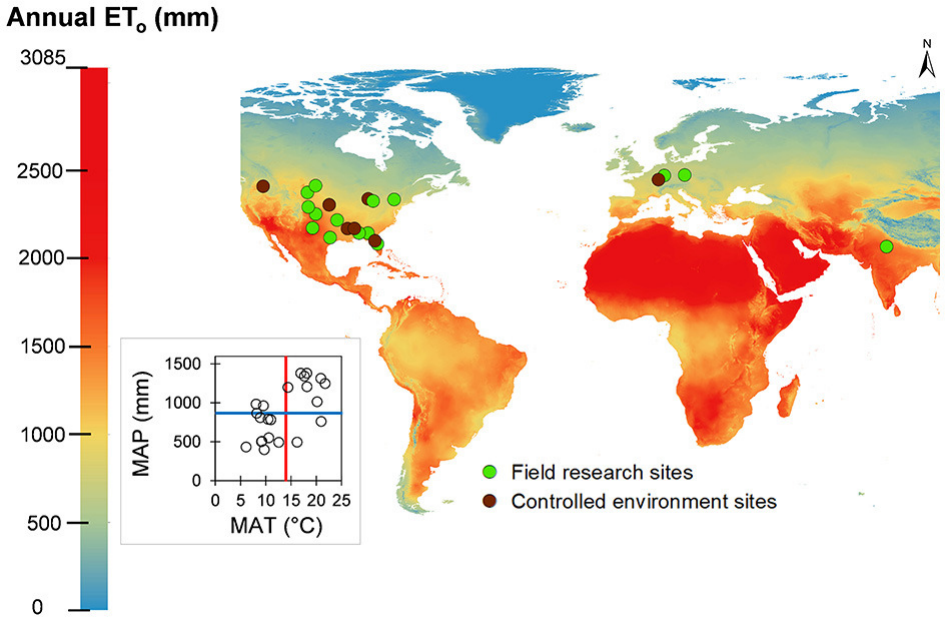
Geographical location and environment (field: green dots; greenhouse: dark red dots) of the 23 studies included in the systematic review, shown over a map of long-term annual grass reference evapotranspiration (ET_o_). The inset shows the mean annual precipitation (MAP) and mean annual temperature (MAT) space for the experimental sites. The horizontal blue and vertical red lines represent the average of MAP and MAT across all locations, respectively.

Among the 23 publications included in this review, a total of 34 weed species were investigated, among which 79% (*n* = 27) were broadleaf and 21% (*n* = 7) were grass weed species. The majority (83%) of the experiments were carried out in North America (specifically, the US, *n* = 19) with the exception of three (13%) in Europe (Czech Republic; Pivec and Brant, 2009, France; Lucero et al., 2000, and Germany; Lopes et al., 2004) and one (4%) in Asia (India; Pandey et al., 2003) (Figure 2). Twelve studies were conducted in a controlled environment (greenhouse), 10 in field conditions, and one in both under a controlled environment and field conditions (Figure 2).

**Figure 3:**
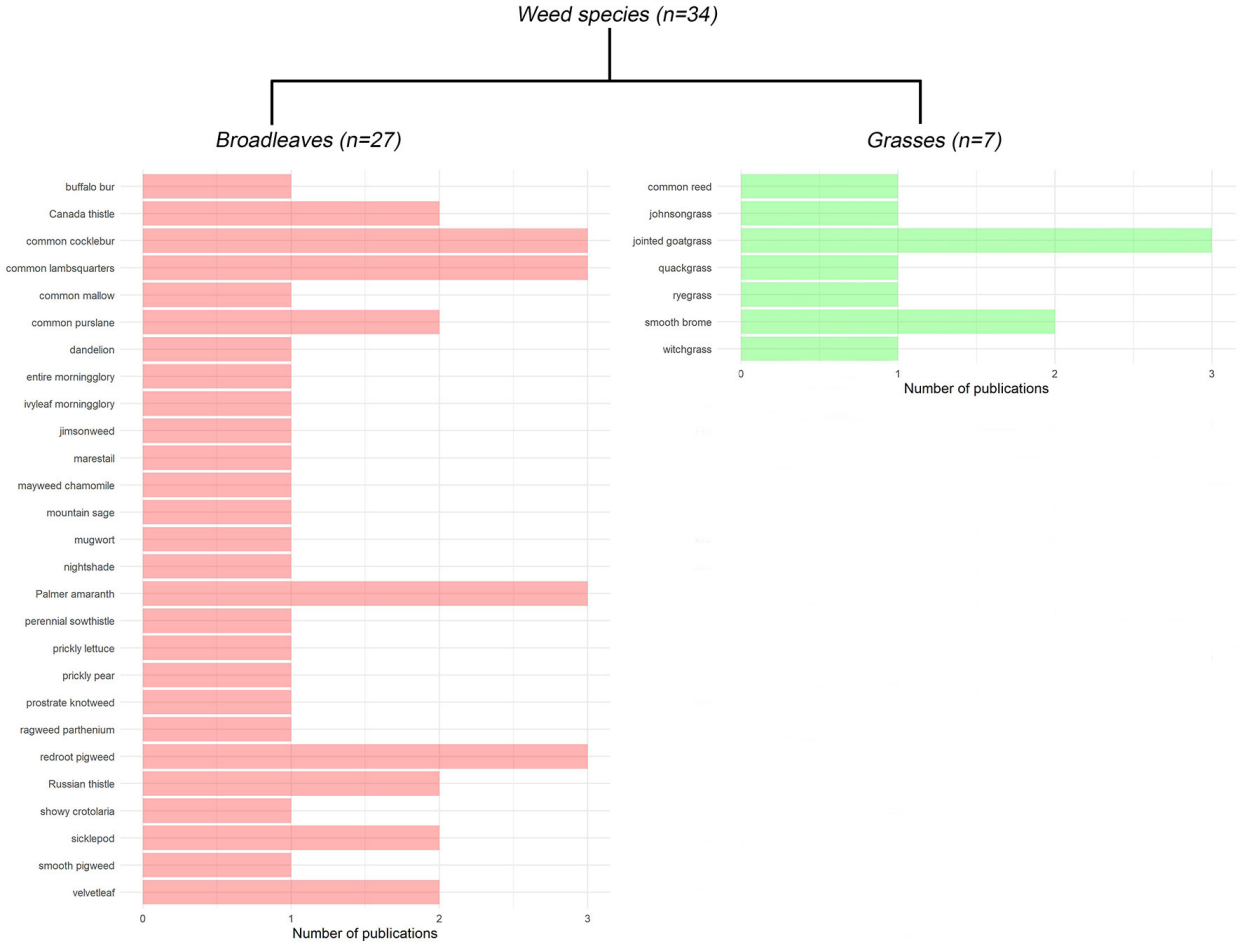
The total number of publications reporting the water use (WU) of broadleaf and grass weed species.

Among the 34 weed species investigated for their WU characteristics, four broadleaf weeds, such as common cocklebur (*Xanthium strumarium* L.), common lambsquarters (*Chenopodium album* L.), Palmer amaranth (*Amaranthus palmeri* S. Watson), and redroot pigweed (*Amaranthus retroflexus* L.); and one grass weed, the jointed goatgrass (*Aegilops cylindrica* Host) were studied from three different publications, the maximum of any species studied (Figure 3). The majority of other weed species, specifically, 67% (*n* = 18) of broadleaf weeds and 71% (*n* = 5) of grass weeds were studied once, with the exception of Canada thistle [*Cirsium arvense* (L.) Scop.], common purslane (*Portulaca oleracea* L.), Russian thistle (*Salsola tragus* L.), sicklepod [*Senna obtusifoli*a (L.) H.S. Irwin & Barneby], and velvetleaf (*Abutilon theophrasti* Medik.) among broadleaves, and smooth brome (*Bromus inermis* Leyss.) among grasses, were investigated twice.

**Figure 4:**
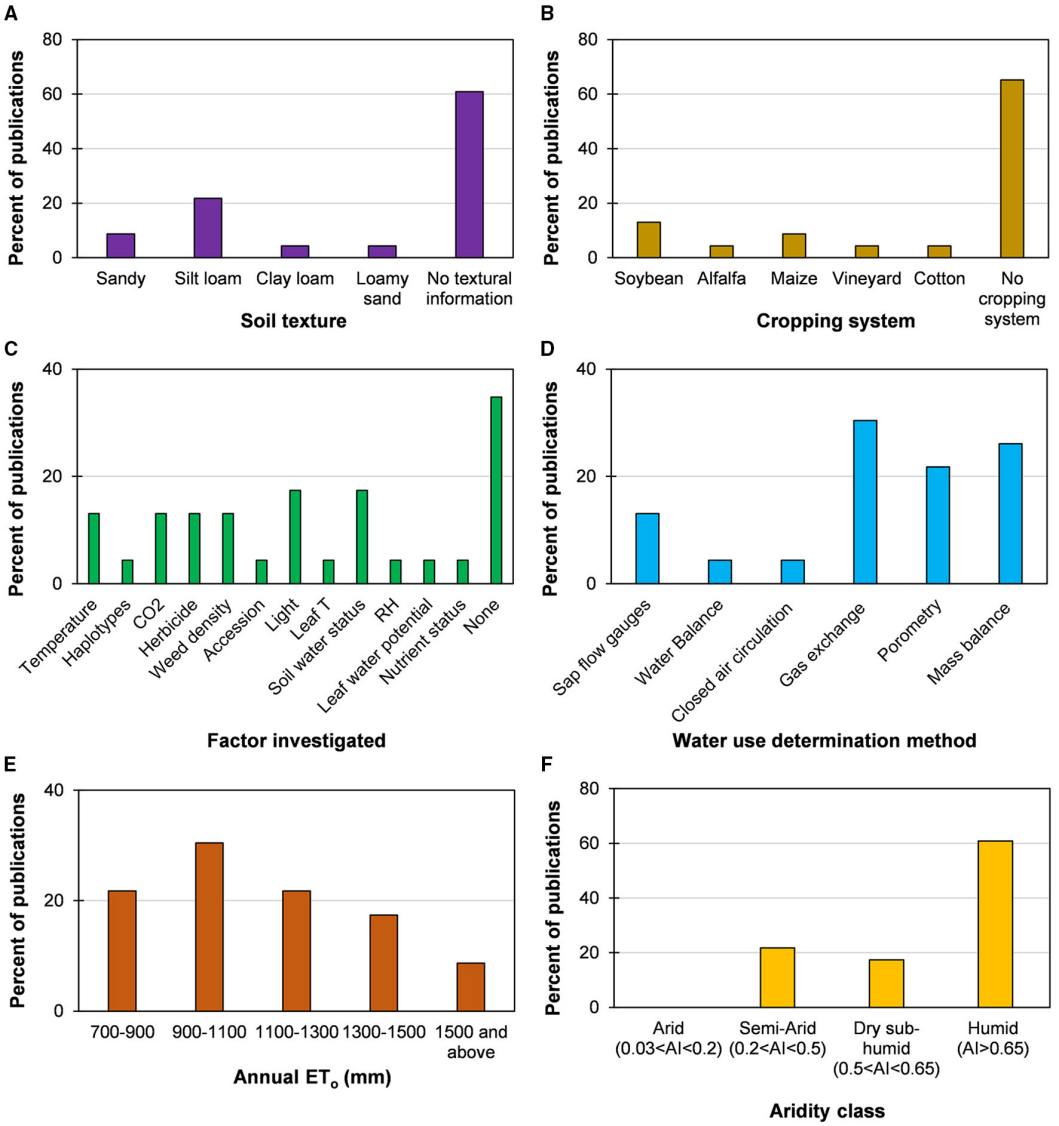
Percentage of publications that present weed water use (WU) in various classes of **(A)** soil texture, **(B)** cropping system, **(C)** factors investigated for the impact on WU, **(D)** method used to determine WU, **(E)** annual evapotranspiration (ET_o_), and **(F)** aridity index (AI).

Across all of the experimental investigations, the long-term (1981–2010) mean annual precipitation (MAP) varied from 399 mm to 1,382 mm, with a global mean of 868 mm. Similarly, long-term mean annual average air temperature (MAT) varied from 6 to 25°C, with a global mean of 14°C. Significant variability existed in MAT and MAP across the experimental locations, which is evident from the points (representing locations) in all the four quadrants of the MAP-MAT space (inset in Figure 2). Daytime and night-time temperatures ranged from 32 to 13°C and 19 to 0°C, respectively, with a global mean of 20 and 8°C, respectively. VPD, which is an indicator of atmospheric dryness, varied from 0.43 to 1.92 kPa, with a global mean of 0.80 kPa. ET_o_ represents a combined effect of all meteorological influences, representing the mean evaporative demand at the experimental locations (base layer in Figure 2). The long-term mean, ET_o_ at the experimental sites varied from 701 mm to 1,830 mm, with a global mean of 1,139 mm. The fact that ET_o_ varied 2.6-fold across the experiments included in this review underscores the role of geographical and spatial heterogeneity in WU assessments. The greatest proportion of studies were conducted in locations with ET_o_ between 900 and1,100 mm, followed by 700–900 mm, 1,100-1,300 mm, 1,300–1,500 mm, and 1,500 mm or more (Figure 4E). If precipitation received at these experimental sites is factored *via* the AI, we observed that most of these studies lie in humid regions, followed by semi-arid and dry-sub-humid regions, with no studies conducted in the arid regions (Figure 4F).

In addition to climatic conditions, the experimental studies also varied by soil texture (Figure 4A), cropping system (Figure 4B), factors investigated (Figure 4C), and method of WU determination (Figure 4D). Most of the studies in the reviewed literature did not include any information on the soil types and cropping systems investigated for weed WU. The soil types that were more subjected to weed WU investigations were silt loam, sandy, loamy sand, and clay loam soils. Weed WU was studied within the stands of soybean, maize, alfalfa, cotton, and in vineyards. A reasonable proportion of these studies was not directly intended to quantify WU for various weed species *per se*; rather, they quantified WU as a target variable as affected by management, environment, and resource availability conditions. However, given the limited direct focus on weed WU quantification, these investigations can be useful sources of WU data under altered conditions.

Around 60% of the publications did not include information about investigating the effects of external factors on weed WU. Among the research that attempted to investigate weed WU relations affected by external factors, the most popular factors were environmental [T, CO_2_, light, and relative humidity (RH)], resource constraints (soil water availability, leaf water potential, and nutrient availability), and management decisions (herbicides and weed density), which were also investigated across multiple studies. Among the other factors investigated were haplotypes, accessions, and leaf temperature. Several determination methods were used depending on which WU metric was intended to be measured. The most popular method was gas exchange, followed by mass balance, porometry, sap flow gauges, water balance, and air circulation through a closed chamber (Figure 4D).

### Weed WU and Its Drivers

**Figure 5:**
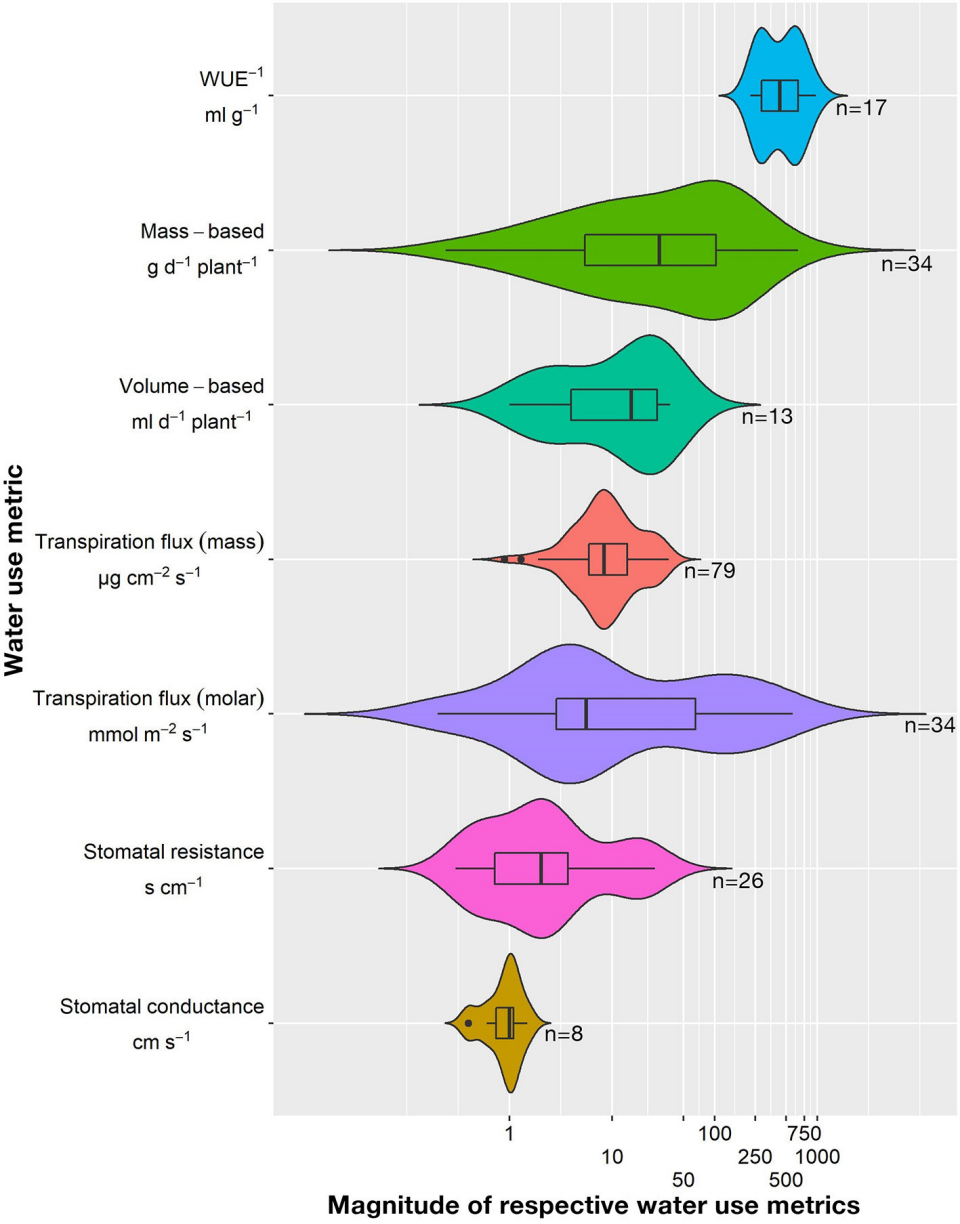
Violin plot with overlay of boxplots of eight water use (WU) estimates reported in the literature. The outline of the violin plot represents the distribution shape of the data. The x-axis of the violin plot has been transformed to log_10_ scale for better visualization. The bold black central line of the boxplot represents the median, the box represents the interquartile range, and the whiskers extend up to the minimum and maximum values excluding the outliers.

Water use of weeds is measured either individually or in cropping systems and/or along with several other experimental factors at variable growth stages and reported as eight different WU metrics. These metrics summate to 226 individual WU observations, arranged alphabetically as per common weed name in Supplementary Table 4. The WU estimates have bimodal or multimodal distribution with high variability and spread (Figure 5). Depth-based WU (coefficient of variation or CV of 2%), stomatal conductance (CV of 36%), water usage effectiveness, (WUE) WUE^−1^ (CV of 45%), mass-based transpiration flux (CV of 62%), and volume-based WU (CV of 81%) have relatively lower variability (CV < 100%) than stomatal resistance (CV of 143%), mass-based WU (CV of 152%), and molar-based transpiration flux (CV of 201%). Overall, the stomatal conductance was the most consistent and evenly distributed (median: 1.0) estimate, with the smallest mean (0.9) and spread (1.1), while WUE^−1^ was the largest variable estimate with the largest mean (484) and spread (753).

The distribution and variability observed in the WU magnitudes stem from the following factors: (i) a wide panel of included weeds, and (ii) contrasting experimental conditions, including weather, crop systems, soils, experimental factors, and levels. This WU variability is evidence for the control of WU by various drivers that can vary from one field to another. These factors are roughly categorized into environmental (temperature, CO_2_, light, and RH), resource constraints (soil water availability, leaf water potential, and nutrient availability), management decisions (herbicide control), and weed-crop competition (crop-weed proximity and density).

The impacts of climate change have been a major focus of weed WU research globally. Overall, the transpiration rates of weed species mostly increase with the increasing temperature, leading to a decline in their WUE (Stutte and Weiland, 1978; Gealy, 1989; Zollinger and Kells, 1991; Pandey et al., 2003; Prince et al., 2018). Transpiration of jointed goatgrass (*Aegilops cylindrica* Host) increased progressively as temperature increased from 10 to 40°C (6.5-fold or 550% increase) at 5°C intervals. Interestingly, transpiration increased despite compensating for continuous increase in diffusive resistance over 20°C, which was intended to reduce Transpiration (Gealy, 1989). Similarly, perennial sowthistle (*Sonchus arvensis* L.) had a 2.4-fold (143%) and 16.2-fold (1517%) increase in transpiration at 30°C (day)/25°C (night) compared to 20/15°C and 10/5°C temperatures, respectively (Zollinger and Kells, 1991). Ragweed parthenium (*Parthenium hysterophorus* L.) transpiration can increase two-fold at 25°C compared to 7°C, at 35°C compared to 25°C, and three-fold at 47°C compared to 35°C (Pandey et al., 2003). Weed species-specific physiology dictates the temperature impacts on transpiration; for instance, Stutte and Weiland (1978) found a significant rise in transpiration in two of six weed species [1.7-fold (68%)] for common cocklebur (*Xanthium strumarium* L.) and 2.3-fold (129%) for Palmer amaranth (*Amaranthus palmeri* S. Watson) when temperature was increased from 28 to 35°C. Additionally, multiple accessions of the same weed species can behave dissimilarly: for example, transpiration in nine accessions of jointed goatgrass ranged between 6.7 and 11.7 μg H_2_O cm^−2^ s^−1^ at the advanced-tillered stage (Gealy, 1988).

Carbon dioxide effects on weed transpiration are mainly studied in interaction with temperature (Patterson and Flint, 1982; Pandey et al., 2003; Prince et al., 2018). In general, if not masked by higher temperature effects (Prince et al., 2018), elevated CO_2_ reduces transpiration and stomatal conductance and hence, results in higher WUE (Patterson and Flint, 1982; Pandey et al., 2003). On the contrary, higher temperatures can also increase transpiration under elevated climate conditions, overruling the effect of increased CO_2_ as observed in the case of common reed [*Phragmites australis* (Cav.) Trin. ex Steud.] haplotypes (Prince et al., 2018).

Transpiration generally shows gradual increase with increasing irradiance (Gealy, 1989; Gealy et al., 1991; Zollinger and Kells, 1991). Transpiration of jointed goatgrass and mayweed chamomile (*Anthemis cotula* L.) nearly doubled as photosynthetic photon flux density (PPFD) increased from 125 to 1850 and 50 to 1800 μE m^−2^ s^−1^, respectively (Gealy, 1989; Gealy et al., 1991). However, transpiration of perennial sowthistle (*Sonchus arvensis* L.) increased more than threefold when PPFD increased from 285 to 1015 μE m^−2^ s^−1^(Zollinger and Kells, 1991). Increasing RH reduces transpiration (Chow et al., 1966) and hence increases WUE (Pandey et al., 2003). With the established importance of temperature, RH, and irradiance, compound variables that report evaporative capacity and demand, such as VPD and ET_o_ should be investigated for their relationships with transpiration, which is currently a significant knowledge gap.

Environmental conditions can also dictate the availability of resources available for vegetation growth and development, indirectly influencing resource uptake in resource-limited scenarios. Limited water availability is a major constraint in semi-arid and arid regions and results in water stress conditions for crops and for weeds as well. When soil water deficit was represented using soil water potential, transpiration in perennial sowthistle (*Sonchus arvensis* L.) reduced by 1.8- fold (43% decrease) at−1 bar than that at 0 bar (Zollinger and Kells, 1991). Similarly, when soil water deficit is represented using leaf water potential, transpiration in the perennial ryegrass (*Lolium perenne* L.) is reduced by 2.6-fold (61% decrease) at−1 MPa than that at 0 MPa (Lucero et al., 2000). Nutrient availability also affects the growth and vigor of weeds and also the transpiration patterns. Weeds also lose gaseous N along with transpiration, which was found to be more sensitive to environmental perturbations than transpiration itself (Stutte and Weiland, 1978).

Management decisions, such as herbicide control and crop-weed competition characteristics, such as crop-weed proximity, density, and time of emergence play an important role in influencing WU in weeds. Effective herbicide application should reduce weed WU over a period of certain days and allocate additional water for the crop. A major knowledge gap lies in understanding how relieving weed pressure (*via* herbicides) in a crop system alters the soil water balance and water uptake by the crop and ameliorates water stress, especially in semi-arid to arid dryland agroecosystems. In any given weed-crop system, partitioning of available water to weed and crop components should be quantified for a complete assessment of water and carbon pools. Water uptake by weeds in irrigated systems is even more concerning, as it involves a direct investment of irrigation water and the associated pumping/diversion expenditure. Mausbach (2021) investigated ET of Palmer amaranth in corn, soybean, and fallow under center pivot and subsurface drip irrigation and found that irrigation method affects Palmer amaranth ET early in the growing season, but crop system and the progression of plant growth with available water have a greater effect on ET than the irrigation method later in the growing season.

An increase in weed density or decrease in weed distance from the crop can dramatically reduce crop leaf water potential, turgor pressure, stomatal conductance, photosynthesis, yield, and WUE (Stuart et al., 1984; Massinga et al., 2003; Berger et al., 2015). A single Palmer amaranth plant can compete with and induce water stress for a crop plant at up to 4 m distance (Berger et al., 2015). While WU of weeds is expected to initially increase with weed density, the rate might diminish after the attainment of a certain density due to mutual shading of the weed plants (Massinga et al., 2003; Berger et al., 2015). To better evaluate the relationship between weed density and WU, water should be maintained as a limiting resource, or else irrigation (Massinga et al., 2003) and excessive rainfall (Berger et al., 2015) would not allow WU dynamics to be revealed at different weed densities. Similarly, weed density impacts on crop water potential, solute potential, or turgor pressure will be absent if water is not limited (Stuart et al., 1984). Conclusively, the importance of these drivers is amplified in the scenarios of limited water, including drought episodes, high evaporative demand and dryland, or limited irrigation systems.

### What Is Challenging to Quantify Weed WU?

The principles, techniques, and models used for WU quantification in vegetation have mainly been intended for and parameterized for uniform, managed, and homogenous systems, such as agricultural systems. In many of these agricultural systems, vegetation characteristics, such as plant density, canopy structure, vigor, canopy coverage, and plant height can be considered as relatively uniform, which allows for the straightforward application of measurement techniques as well as modeling frameworks. Moreover, the general acceptance and recognition by stakeholders about the potential for economic profitability associated with agricultural water management has led to the priority of crop WU quantification.

Agricultural systems, however, are only one component of global vegetation, and they largely differ in their characteristics from other vegetation types. Nonagricultural systems, such as deserts, forests, and riparian vegetation are characterized by a large heterogeneity in all of the aspects that managed the homogenized agricultural systems. Weed systems are one such heterogeneous class, but they are often even more complex because of the coexistence and competition with profit-oriented agricultural systems. Due to the low predictability of weed growth, structure, density, ground coverage, and other factors, the radiative and aerodynamic processes are of a somewhat wicked nature: it is challenging to define, parameterize, and represent their spatial behavior. The coexistence of weeds and agricultural crops makes this process even more challenging, owing to the increased complexity of resource trade-offs (light, water, carbon, and nutrients), weed inhibition and control strategies, and irrigation and nutrient management of the main crop.

### Limitations of the Existing Research and Future Research Directions

Water use data from any vegetation system may contain biases from suboptimal experimental design, measurement technique, management, data processing, definition inconsistencies, model structure and parameterizations, and interpretation (Allen et al., 2011). Even the WU estimates of agricultural crop can seldom be affected by these biases, though their prevention is ensured through a critical system established *via* training, peer-review, and discussions in the agricultural water management, soil and water resources engineering, biometeorology, and irrigation science and engineering communities. The most important safeguard against these biases include sufficient and proper documentation and description of critical definitions, variables, measurements, and metadata that accompany the WU estimates, along with the best practices for conducting and reporting WU research.

This systematic review revealed several of these weaknesses in the literature, which were a consequence of not subscribing to the best practices of measuring, estimating, and reporting WU data. The following sections expand on the limitations in the literature and seek to inform the weed scientists on how to avoid these pitfalls that undermine the significance, application, and reliability of datasets.

#### Heterogeneity of WU Metrics Reported in the Literature

Throughout the literature, there exists a significant heterogeneity on what terminology/definitions to employ when investigating the WU characteristics of weeds. Synthesis of robust WU estimates across global research efforts necessitates that there should be a strong consensus on (i) which variables sufficiently represent weed WU for effective applications; (ii) what definitions/formulations should be used to quantify/estimate these variables; and (iii) what vegetation footprint and temporal resolution are desirable for effective application.

Amongst the literature, WU was represented *via* kg d^−1^ of sap flow (Pivec and Brant, 2009), sap flow normalized by leaf area (Berger et al., 2015), cm d^−1^ (Massinga et al., 2003), transpiration reported as T m^−2^ of soil d^−1^ (Lopes et al., 2004), ml plant^−1^ (Lucero et al., 2000), and gas exchange-based instantaneous leaf-level T (Stuart et al., 1984; Munger et al., 1987; Gealy, 1988; Trimmer and Linscott, 1990; Zollinger and Kells, 1991), ET based on mass balance (Shantz and Piemeisel, 1927; Dillman, 1931), and others.

From a systems-level water management perspective, WU in agronomic crops is referred to as crop ET, which is represented as the depth of water during a period, e.g., mm d^−1^. Agronomic WU research usually reports seasonal total ET (mm) data under a set of soil, weather, and management regimes. To study the impacts of soil water availability, water consumption of crops as affected by weeds, or *vice versa*, it is critical that weed WU should be reported at consistent spatial and temporal resolutions as well as in the same physical quantities. Vegetation WU is driven by plant growth and weather characteristics, and thus is subject to trends and variability within the growing season. Therefore, to truly characterize WU, it is vital to compute high temporal resolution (daily, weekly) of weed ET or weed-crop system ET. From a producers' standpoint, weed and crop WU should be physically comparable to allow for the effective visualization of the water penalty of weeds for an agricultural operation. Crop WU is extensively reported as depth of water during a period *via* university extension, crop consultants, and private irrigation service providers, and so weed WU should be reported in the same fashion.

Alternatively, if other physical quantities are to be used for specific purposes, sufficient detail should be provided to allow for interconversions (Kukal and Irmak, 2019, 2020). For example, Jones et al. (1997) presented a sap flow in common cocklebur and sicklepod [*Senna obtusifoli*a (L.) H.S. Irwin & Barneby] on several bases: per individual plant, per leaf area, and per ground area, which allows for normalized comparisons across the two species and their biophysical features.

#### Requirements for Effective Communication and Interpretation of WU

Reporting of experimental investigations in the field and controlled conditions to measure WU parameters should be considerate of certain fundamental requirements and best practices necessary for the effective measurement, reporting, and interpretation of WU. Adherence to the best practices ensures that the discoverability of findings is maximized, reuse of the data is efficient, and applications of data to global ecosystems is facilitated.

##### (a) Relevant Soil Information

In our review, it was a common occurrence for researchers to ignore or fail to mention soil properties that are critical to comprehensively understand the soil-plant-atmospheric relationships, e.g., saturation point, field capacity, permanent wilting point, water holding capacity, particle size distribution, infiltration rate, residue cover, etc. WU estimates, especially ET, cannot be interpreted fairly across dissimilar media if this information is not measured. Thus, it is recommended to effectively sample soil media prior to measuring and reporting ET rates. This may mean grid-based depth-specific soil sampling and analyses for field investigations and the characterization of standardized potting mixes, since most of these properties are largely static. Like any sound experimental study, all external conditions that could affect weed water acquisition and use should be explicitly characterized and accounted for. Spatial differences in soil series and terrain slope across treatments should be accounted for using experimental blocks.

##### (b) Evaporative Demand

Water use estimates reported in the literature are not useful, interpretable, or transferable if they lack accurate evaporative demand information. Access to evaporative demand information aids in understanding the true WU of vegetation after accounting for how “thirsty” the environment was where WU was measured. In our review, none of the controlled environment-based investigations and few of the field-based investigations reported evaporative demand data. When evaporative demand was reported, it was seldom estimated using standard definitions and techniques.

The ASCE reference ET is the standardized metric for reporting the evaporative demand data (Walter et al., 2000; Jensen and Allen, 2016). Depending on the hypothetical reference surface, this metric can be grass-reference evapotranspiration (ET_o_) or alfalfa-reference evapotranspiration (ET_r_). Supplementary Table 2 lists ET_o_ at each of the experimental sites across the included literature. Determining ET_o_ or ET_r_ requires a standardized agricultural weather station that records T, RH, solar radiation, and wind speed. A weather station at the experimental site can be set up or a close-by weather station can be selected from public weather networks, e.g., six regional climate centers within the NOAA's National Centers for Environmental Information (NCEI).

This need becomes especially critical if the WU data are measured and reported from environments and temporal periods that are different from where they are intended to be applied/utilized. Direct comparisons of ET rates and depths with ET determined at other locations or time periods is a malpractice, unless accompanied by ET_o_ or ET_r_ information. For example, the 69.6 cm of ET in Palmer amaranth reported by Massinga et al. (2003) at Kansas, USA will not be very useful for a decision-maker in need of Palmer WU data located in Michigan, USA. The ET of Palmer amaranth across the two sites will vary substantially, as the latter site has roughly half the annual ET_o_ (796 mm) than the former (1,425 mm). If sampling period of total ET_o_ information is reported along with the ET data, the user can calibrate the ET for effective decision-making at the target location. Thus, ET_o_ corresponding to the WU measurement location and period is central and indispensable to WU reporting. An effective way to achieve transferability and to account for space-time weather variability is to normalize the ET estimates/measurements using ET_o_ and transforming the ET depths into crop coefficients or fraction of reference ET (Allen et al., 2011).

##### (c) Weed Characteristics

Various aspects of vegetative (weed) conditions dictate WU, such as leaf area index (LAI), phenological stage (green-up, maximum cover, flowering, and senescence), plant density, plant height, fraction of ground cover, and others. It is recommended that adequate weed condition information should be reported along with the WU estimates. Ideally, the WU measurements should be conducted for the entire active growth period of the weed (emergence until end of senescence), and thus the progression of vegetation characteristics during this time should be reported (e.g., LAI vs. time).

Our review suggests that most of the studies reported weed growth at measurement in one form or another. However, the most common reporting scale was days after planting (DAP), which is not transferable across years or locations. Plant (weed) phenology is sensitive to heat available for growth (often represented by growing degree days or thermal time), which varies from year to year and location to location. Thus, it is recommended that weed growth and WU should be represented using phenological stages, such as Biologische Bundesanstalt, Bundessortenamt und CHemische Industrie (BBCH) or cumulative heat (growing degree days) accumulation until the measurement period, enabling effective transferability across space and time (Kukal and Irmak, 2019). Moreover, since both crop and weed canopy characteristics together affect the surface energy balance and dictate resource competition in the crop-weed system, crop conditions should be reported as well.

##### (d) Soil Water Status

Both weed and crop roots compete for soil water extraction, their water uptake being a function of water availability. Water stress conditions, defined by a fraction (referred to as manageable allowable depletion or MAD) of plant-available water in the root zone profile, can impair water uptake and plant productivity. Crops are managed (in irrigated production) so that the available soil water remains sufficiently above MAD *via* irrigation scheduling tools. Under rainfed conditions, the lower extreme of available water is only a function of precipitation, and significant drought episodes can occur. Weeds are assumed to be relatively less sensitive to water than cash crops, and hence should maintain greater vigor during stressed conditions compared to the main crop. Thus, weed-crop ET estimates lacking soil water status information fail to represent whether the reported water uptake was under water-sufficient or stressed conditions. The ET measured when the available water is below the soil water stress threshold will be lower compared to when an irrigation or precipitation event has replenished the root zone profile to field capacity.

Ideally, soil moisture conditions (volumetric water content) at incremental depths in the root zone should be reported throughout the active growing season, along with irrigation and precipitation events. Consequently, total soil water in the root zone (mm) should be calculated and presented in relation to the upper (field capacity) and the lower (permanent wilting point) limits of the soil water storage. This can be accomplished using soil moisture sensing technologies commercially available (SU et al., 2014; Sharma et al., 2021).

##### (e) Details of Sampling and Measurements in WU Determination

Given the uncertainties and errors associated with WU measurements/sampling techniques, it is critical that the reader/user is well versed in the minutiae of the procedures followed when measuring WU. The choice of certain techniques and protocols within the technique can significantly impact WU assessments. These include parameters, assumptions, instrumentation, sampling design, frequency (spatial and temporal), calibration procedures, etc.

The review suggests that gas exchange, porometry, mass balance, sap flow gauges, and water balance are the dominant WU techniques. Recently, Ely et al. (2021) proposed a reporting format for leaf-level gas exchange data and metadata to facilitate data consistency, application, and harmonized synthesis. This format can comfortably be extended to weed systems, as all of the reporting parameters and definitions apply to all plant functional types. Sap flux-measured WU should be accompanied with the number of plants sampled, selection criteria within a weed stand, scaling procedures from a plant to ground area basis, calibration procedures (if any), measurement frequency, height of sensor orientation, insulation method, etc. Water balance-derived WU estimates should be accompanied with soil water holding properties, accuracy assessment, and calibration functions (if any) for soil moisture-sensing technology used, rooting zone depths, number of sensing locations, sensing of incremental depths within the root zone, treatment of all water balance components (deep percolation, runoff, irrigation, precipitation, soil water storage, and upward flux), surface cover characteristics, pedo-transfer functions used, temporal measurement/observation frequency, etc.

#### Measurement and Modeling of Crop-Weed System Water Use

**Figure 6:**
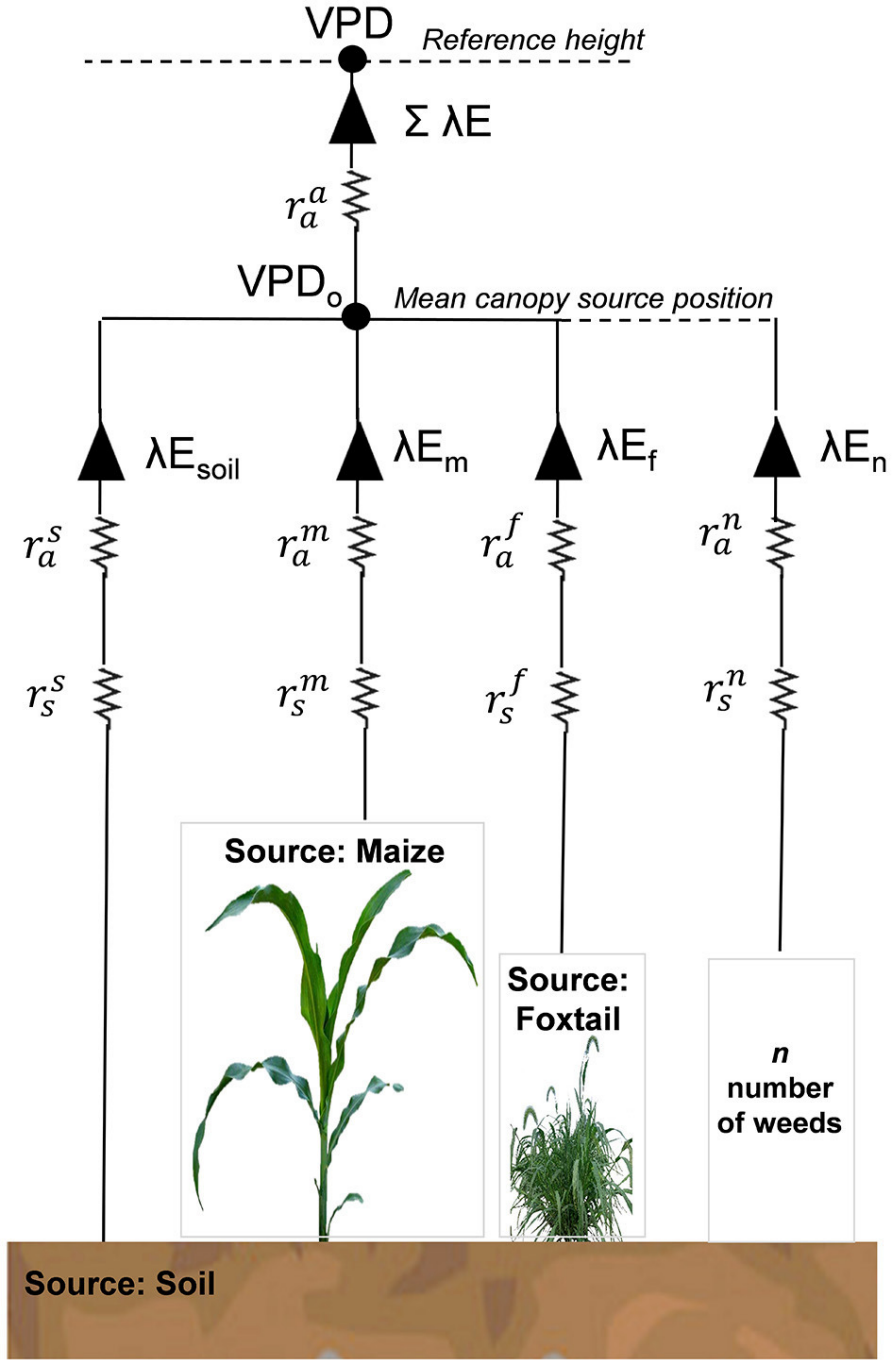
A schematic representation of the evapotranspiration (ET) from a weed-crop system. In this particular example, constituents *i* are soil (superscript *s*), maize crop (superscript *m*), and foxtail species (superscript *f*), with the possibility of extension to *n* number of weed species. Each species has its own canopy resistance ($r_{s}^{i}$) and boundary layer resistance ($r_{a}^{i}$). Each constituent *i* has its component latent heat fluxes (evaporation for soil component), λE_*i*_, that converge within the mixed canopy where the vapor pressure deficit is *VPD*_*o*_. The total latent heat flux λE mixes with the air at reference height *via* the aerodynamic resistance, $r_{a}^{a}$.

In a commercial agricultural field, multiple weed species can exist adjacent to the main cash crop, resulting in a complex mixed plant community. To understand the ET of the entire mixed plant community system, it is critical that each component of the system should be viewed as an evaporation source. Generally defined, *n* plant species exist, and there are (*n* + 1) sources of heat and vapor, including soil as a source. For example, a weed-crop system, as illustrated in Figure 6, should consist of a main crop (maize) and a weed species (foxtail; *Setaria* spp.), and can be extended to *n* weed species. Following Wallace (1997), total latent heat flux (λ*ET*) from such a weed-crop community is expressed as follows:


(1) $$\begin{array}{r} \lambda ET=\overset{n+1}{\underset{i=1}{\sum }}C_{i}PM_{i} \end{array}$$

where λ is the latent heat of vaporization of water; C_*i*_ is a coefficient for each constituent species *i*; and *PM*_*i*_ are terms similar to the Penman-Monteith formula applied to closed canopies of each constituent species, *i*. These terms are defined as follows:


(2) $$\begin{array}{r} PM_{i}=\frac{\Delta A+\left\{\rho c_{p}VPD-\Delta r_{a}^{i}\left(A-A_{i}\right)\right\}/\left(r_{a}^{a}+r_{a}^{i}\right)}{\Delta +\gamma \left\{1+r_{s}^{i}/\left(r_{a}^{a}+r_{a}^{i}\right)\right\}} \end{array}$$

(3) $$\begin{array}{r} C_{i}={\left(1+\frac{\sum_{j\neq \;1}^{n}1/R_{j}}{\frac{1}{R_{i}}+\frac{1}{R_{a}}}\right)}^{-1} \end{array}$$

where Δ is the rate of change of saturated vapor pressure with temperature; A is the total energy available to the weed-crop system; ρ and *c*_*p*_ are the density and specific heat (at constant pressure) of the air; γ is the psychrometric constant; VPD is the vapor pressure deficit above the canopy; $r_{a}^{i}$ is the aerodynamic resistance to water vapor transfer from the canopy to a point in the air around the canopy where the vapor pressure deficit is *VPD*_*o*_; A_*i*_ is the available energy for each species; $r_{a}^{a}$ is the aerodynamic resistance between the in-canopy mixing point (VPD_0_) and the reference height (VPD); and $r_{s}^{i}$ is the canopy resistance of each species *i*, calculated from their stomatal resistances and leaf area indices.

*R*_*a*_ and *R*_*i*_ are defined as follows:


(4) $$\begin{array}{r} R_{a}=\left(\Delta +\gamma \right)r_{a}^{a} \end{array}$$

(5) $$\begin{array}{r} R_{i}=\left(\Delta +\gamma \right)r_{a}^{i}+r_{s}^{i} \end{array}$$

the available energy for each species is given by the following equation:


(6) $$\begin{array}{r} A_{i}=f_{i}R_{n} \end{array}$$

and the available energy for the soil component is given by the following equation:


(7) $$\begin{array}{r} A_{s}=\left(1-\overset{n}{\underset{i=1}{\sum }}f_{i}\right)R_{n}-G \end{array}$$

where G is the soil heat flux. It is also critical to know the above-canopy climatic conditions to calculate the total ET from the weed-crop canopy. *VPD*_*o*_ can be calculated as follows:


$$\begin{array}{l} VPD_{o}=VPD+\left\{\Delta A-\left(\Delta +\gamma \right)\lambda E\right\}r_{a}^{a}/\rho c_{p} \end{array}$$

Latent heat flux from individual component-species (λE_*i*_) is calculated as below:


$$\begin{array}{l} \lambda E_{i}=\Delta \frac{f_{i}R_{n}+\rho c_{p}VPD_{o}/r_{a}^{i}}{\Delta +\gamma \left(1+r_{s}^{i}/r_{a}^{i}\right)} \end{array}$$

where *f*_*i*_ is the fraction of the above canopy net radiation (*R*_*n*_) that is intercepted by canopy *i* (crop or weed).

#### Future Research Directions in the Weed Water Use Domain

Based on the current knowledge gaps in the literature and the potential usefulness of WU-related information for stakeholders, we propose the following suggestions for research directions at the intersection of crop and weed management and water resources management:

1. Develop “weed coefficients” for major weed species for different phenological stages following the FAO crop coefficient concept (Allen et al., 1998). This will allow the WU information to be transferable across environmental conditions.
2. Quantify relative rates of weed and crop WU and their evolution during the growing season, especially under different evaporative demand scenarios.
3. Investigate the impact of weed ET on modifying the surface energy balance and microclimate for agronomic crops, *via* alterations in radiative energy budget, soil moisture, and ground coverage area.
4. Investigate how WU dynamics of the main crop are affected by *n* number of weed species with varying densities and proximities.
5. Couple (a) WU-related impacts and (b) yield penalties from weeds on agronomic crops to assess their impacts on the WUE of the crop.
6. Develop upper and lower water stress baselines (Gardner et al., 1992; Irmak et al., 2000; Han et al., 2018) to determine the weed productivity response to water for different weeds and for their control.
7. Bridge and harmonize WU estimates across the leaf-level, whole-plant level, community-level, and ecosystem-level.
8. Study how the WU of individual and crop-weed system respond to irrigation methods (sprinkler vs. surface vs. micro-irrigation) and irrigation management regimes (e.g., deficit vs. full irrigation).
9. Partition crop-weed system ET into E and transpiration components using measurements and modeling.
10. Study root-zone soil water extraction and the contribution of various root layers to total soil water extraction for various weed species (Djaman and Irmak, 2012; Irmak et al., 2014; Nielsen and Vigil, 2018; Kukal and Irmak, 2020), and compare and contrast them among crop water extraction to evaluate crop-weed competition for limited soil water reserves.
11. Investigate interactions of weed and weed-crop system WU with various factors that can vary in commercial fields and environmental changes projected for local ecosystems.

## Conclusion

The systematic review suggested that weed WU research has resulted in sporadic estimates of WU metrics across weed species, locations, environments, soils, and cropping systems. While the limited research information is useful, it fails to accomplish the longstanding and important challenge of quantifying and reporting weed WU in a fair, robust, synthesizable, and transferrable fashion. This is partially due to the fact that research investigations often lack adherence to certain fundamental requirements and the best practices of WU quantification research and its reporting. From the standpoint of agricultural water management, soil and water resources engineering, and irrigation science and engineering, we identify these pitfalls and limitations and offer recommendations for avoiding them. Adherence to these best practices can be instrumental in ensuring a unified and consistent large-scale effort in the weed science community to decipher crop-weed-water interactions and apply them in empirical/mechanistic models of weed-crop competition. We believe that this systematic synthesis achieves unified summarization of available data on weed WU, emphasizes the importance of weed WU quantification, and encourages better formulations, experimental protocols, and reporting practices for the weed science community.

## Data Availability Statement

The original contributions presented in the study are included in the article/Supplementary Material, further inquiries can be directed to the corresponding author/s.

## Author Contributions

MS, MK, and SI: did literature search, conceptualize the concept, and wrote manuscript. AJ: provided guideline and edited manuscript. All authors contributed to the article and approved the submitted version.

## Conflict of Interest

The authors declare that the research was conducted in the absence of any commercial or financial relationships that could be construed as a potential conflict of interest.

## Publisher's Note

All claims expressed in this article are solely those of the authors and do not necessarily represent those of their affiliated organizations, or those of the publisher, the editors and the reviewers. Any product that may be evaluated in this article, or claim that may be made by its manufacturer, is not guaranteed or endorsed by the publisher.

## Abbreviations

A: Total energy available to the weed-crop system
A_*i*_: Available energy for each species
*C*_*i*_: Coefficient for each constituent species *i*
*c*_*p*_: Specific heat (at constant pressure) of air
CV: Coefficient of variation
DAP: Days after planting
ET: Evapotranspiration
ET_o_: Grass-reference evapotranspiration
ET_r_: Alfalfa-reference evapotranspiration
*f*_*i*_: Fraction of the above canopy net radiation (*R*_*n*_) intercepted by canopy *i* (crop or weed)
G: Soil heat flux
g_l_: stomatal conductance
*i*: superscript *i* in Figure 6 is soil (s), maize crop (m), or foxtail weed species (f)
LAI: Leaf area index
MAD: Manageable allowable depletion
MAP: Mean annual precipitation
MAT: Mean annual temperature
ρ: Density (at constant pressure) of air
PM_*i*_: Penman-Monteith evaporation term for closed canopies of each constituent species *i*
PPFD: Photosynthetic photon flux density
RH: Relative humidity
r_l_: Stomatal resistance
*R*_*n*_: Above canopy net radiation intercepted by canopy *i* (crop or weed)
$r_{a}^{a}$: Aerodynamic resistance between the in-canopy mixing point (*VPD*_*o*_) and the reference height (VPD)
$r_{a}^{i}$: Aerodynamic resistance to water vapor transfer from the canopy to a point in the air around the canopy where the vapor pressure deficit is *D_o_*;
$r_{s}^{i}$: Canopy resistance of each species *i*, calculated from their stomatal resistances and leaf area indices
T (°C): Air temperature
T_q_: Sap flow
VPD: Vapor pressure deficit above the canopy
WU: Water use
WUE: Water use efficiency
Δ: Rate of change of saturated vapor pressure with temperature
λ: Latent heat of vaporization of water
λET: Latent heat flux
λE_*i*_: Latent heat flux from individual component species
γ: Psychrometric constant.
