# Supplementary material for: Water Use Characteristics of Weeds: A Global Review, Best Practices, and Future Directions
Source: Front Plant Sci. 2022 Jan 7;12:794090. doi: 10.3389/fpls.2021.794090 (PMC8777227; doi:10.3389/fpls.2021.794090)
Supplement: Supplementary file 1 [file Data_Sheet_1.docx]

**Supplementary Material for the manuscript “Water Use Characteristics of Weeds: A Global Review, Best Practices and Future Directions”**

**Table S1:** List of search terms used for searching relevant primary literature. The search term “Weed” or each weed species name (common and scientific name) was combined with “AND” to include all the constant terms in the second column.

| **“Weed” or weed species** | **Constant terms** |
| --- | --- |
| (“Weed”) | (“Evapotranspiration" OR "Water Use" OR "Water Loss" OR "Sap Flow" OR “Transpiration” OR “Bowen Ratio”) |
| (“foxtail” OR “green foxtail” OR “Setaria viridis” OR “giant foxtail” OR “Setaria faberi” OR “yellow foxtail” OR “Setaria pumila”) |  |
| (“crabgrass” OR “large crabgrass” OR “Digitaria sanguinalis” OR “smooth crabgrass” OR “Digitaria ischaemum” OR “southern crabgrass” OR “Digitaria ciliaris”) |  |
| (“common lambsquarters” OR “Chenopodium album”) |  |
| (“Bromus” OR “downy brome” OR “Bromus tectorum” OR “smooth brome” OR “Bromus inermis” OR “Japanese brome” OR “Bromus japonicus” OR “cheatgrass” OR “rescuegrass” OR “Bromus catharticus”) |  |
| (“Lamium” OR “henbit” OR “Lamium amplexicaule” OR “purple deadnettle” OR “Lamium purpureum”) |  |
| (“bluegrass” OR “annual bluegrass” OR “Poa annua” OR “Roughstalk bluegrass” OR “Poa trivialis” OR “Kentucky bluegrass” OR “Poa pratensis”) |  |
| (“Palmer amaranth” OR “Amaranthus palmeri”) |  |
| (“Italian ryegrass” OR “Lolium multiflorum”) |  |
| (“dandelion” OR “Taraxacum officinale”) |  |
| (“common chickweed” OR “Stellaria media”) |  |
| (“horseweed” OR “marestail” OR “Conyza canadensis”) |  |
| (“waterhemp” OR “Amaranthus tuberculatus”) |  |
| (“Canada thistle” OR “Cirsium arvense”) |  |
| (“johnsongrass” OR “Sorghum halepense”) |  |
| (“morningglory” OR “ivyleaf morningglory” OR “Ipomoea hederacea” OR “pitted morningglory” OR “Ipomoea lacunosa” “tall morningglory” OR “Ipomoea purpurea”) |  |
| (“kochia” OR “Bassia scoparia”) |  |
| (“nutsedge” OR “yellow nutsedge” OR “Cyperus esculentus” OR “purple nutsedge” OR “Cyperus rotundus”) |  |
| (“pigweed” OR “redroot pigweed” OR “Amaranthus retroflexus” OR “smooth pigweed” OR “Amaranthus hybridus” OR “Powell amaranth” OR “Amaranthus powelli” OR “spiny amaranth” OR “Amaranthus spinosus”) |  |
| (“common ragweed” OR “Ambrosia artemisiifolia”) |  |

**Table S2:** List of papers (n = 23) included in the systematic review with their reference IDs, actual references, weeds investigated, experimental locations, annual weather normals (1981-2010), and soil types.

| **Reference ID** | **Reference** | **Weed** | **Experimental site** | **Annual Average Temperature (°C) Normal (1981-2010)** | **Annual Precipitation (mm) Normal (1981-2010)** | **Annual ETo (mm) Normal (1981-2010)** | **Annual mean AI Normal (1981-2010)** | **Annual Mean VPD (kPa) Normal (1981-2010)** | **Soil type** |
| --- | --- | --- | --- | --- | --- | --- | --- | --- | --- |
| A | Shantz and Piemeisel, 1927 | buffalo bur, common cocklebur, common lambsquarters, common sunflower, common purslane, mountain sage, nightshade, prostrate knotweed, redroot pigweed, Russian thistle,  smooth brome | Akron, Colorado, US | 10 | 399 | 1236 | 0.32 | 0.86 | - |
| B | Dillman, 1931 | common lambsquarters, common purslane,  redroot pigweed, Russian thistle,  smooth brome, witchgrass | Mandon, North Dakota, US | 6 | 432 | 906 | 0.48 | 0.58 | - |
| C | Chow et al, 1966 | prickly pear | Lincoln, Nebraska, US | 11 | 785 | 1042 | 0.75 | 0.66 | Coarse sand |
| D | Stutte and Weilanad, 1978 | johnsongrass, entire mornigglory, ivyleaf morningglory, jimsonweed, cocklebur, Palmer amaranth | Fayetteville, Arkansas, US | 14 | 1200 | 1170 | 1.03 | 0.73 | Taloka silt loam (fine, mixed, thermic, Mollic Albaqualfs) |
| E | Patterson and Flint, 1982 | sicklepod, showy crotolaria | Stoneville, Mississippi, US | 18 | 1344 | 1240 | 1.08 | 0.82 | - |
| F | Stuart et al, 1984 | smooth pigweed | Lubbock, Texas, US | 16 | 496 | 1830 | 0.27 | 1.27 | Olton clay loam soil (Aridic Paleustolls) |
| G | Munger et al, 1987 | velvetleaf | College station, Texas, US | 20 | 1016 | 1405 | 0.72 | 1.01 | Weswood silt loam soil (Fluventic Ustochrepts) |
| H | Gealy, 1987 | jointed goatgrass | Washington, US | 9 | 502 | 933 | 0.54 | 0.69 | - |
| I | Gealy, 1988 | jointed goatgrass | Pullman, Washington, US | 9 | 502 | 933 | 0.54 | 0.69 | - |
| J | Gealy, 1989 | jointed goatgrass | Washington, US | 9 | 502 | 933 | 0.54 | 0.69 | - |
| K | Trimmer and Linscott, 1990 | quackgrass | Etna, New York, US | 8 | 986 | 751 | 1.31 | 0.44 | Langford Channery silt loam (coarse loamy, mixed mesic, Aqueptic Fragiudalf |
| L | Zollinger and Kells, 1991 | perennial sowthistle | Shiawassee Co., Michigan, US | 9 | 810 | 796 | 1.02 | 0.49 | - |
| M | Gealy et al, 1991 | mayweed chamomile | Washington, US | 9 | 502 | 933 | 0.54 | 0.69 | - |
| N | Holloway and Shaw, 1996 | ivyleaf morningglory | Mississippi, US | 17 | 1382 | 1181 | 1.17 | 0.77 | - |
| O | Jones et al, 1997 | sicklepod, common cocklebur | Auburn, Alabama, US | 18 | 1382 | 1233 | 1.12 | 0.87 | Marvin loamy sand (fine-loamy, siliceous, ther- mic, Typic Kanhapludults) |
| P | Lucero et al, 2000 | ryegrass | Nancy, France | 10 | 790 | 762 | 1.04 | 0.44 | - |
| Q | Massinga et al, 2003 | Palmer amaranth | Garden city, Kansas, US | 13 | 494 | 1425 | 0.35 | 0.95 | Richfield silt loam (mesic Aridic Argiustoll) |
| R | Pandey et al, 2003 | ragweed parthenium | Kosi, UP, India | 25 | 616 | 1539 | 0.40 | 1.92 | - |
| S | Lopes et al, 2004 | common lambsquarters, Canada thistle, common mallow, dandelion | Johannisberger, Geisenheim, Germany | 11 | 552 | 721 | 0.77 | 0.46 | Deep loamy loess |
| T | Pivec and Brant, 2009 | redroot pigweed, mugwort, Canada thistle, marestail, prickly lettuce | Praha-Suchdol, Prague, Czech Republic ; Červený Újezd, Czech Republic | 9 | 505 | 701 | 0.72 | 0.43 | - |
| U | Berger et al, 2015 | Palmer amaranth | Citra, Florida, US; Georgia, US | 22 | 1249 | 1401 | 0.89 | 0.91 | Arredono fine sand |
| V | Vaughn et al, 2016 | velvetleaf | Lincoln, Nebraska, US | 11 | 784 | 1047 | 0.75 | 0.66 | - |
| W | Prince et al, 2018 | common reed | Gainesville, Florida, US | 21 | 1317 | 1327 | 0.99 | 0.93 | Commercial potting soil (Professional Growing Mix, Sun Gro Horticulture Canada, Agawam, MA, USA) |

**Table S3**. Literature-reported unitary systems of water use metrics and the homogenized unitary system to which they were converted upon compilation.

| **WU metric** | **Unitary systems** | |
| --- | --- | --- |
|  | **Literature-reported** | **Homogenized** |
| Mass-based WU | kg d^-1^ plant^-1^ | g d^-1^ plant^-1^ |
|  | g hr^-1^ plant ^-1^ |  |
| Volume-based WU | ml plant^-1^ | ml d^-1^ plant^-1^ |
| Mass-based Transpiration flux | g dm^-2^ hr^-1^ | μg cm^-2^ s^-1^ |
|  | g cm^-2^ d^-1^ |  |
|  | mg m^-2^ s^-1^ |  |
| Molar-based Transpiration flux | mmol m^-2^ s^-1^ | mol m^-2^ s^-1^ |
