# Supplementary material for: Water Use Characteristics of Weeds: A Global Review, Best Practices, and Future Directions
Source: Front Plant Sci. 2022 Jan 7;12:794090. doi: 10.3389/fpls.2021.794090 (PMC8777227; doi:10.3389/fpls.2021.794090)
Supplement: Supplementary file 2 [file Table_4.docx]

**Table S4:** Studies (n = 23) evaluated in the systematic review, including common and scientific name of the weed species, their troublesome rank as per 2019-20 WSSA national surveys, encoded references, cropping system under study, stage of weed growth at the time of water use (WU) measurement, factors investigated in each study along with their values, WU metric, and actual measured amount of WU.

| **Species (common and scientific name)** | **WSSA troublesome rank*** | **Reference**  **code†** | **Cropping system** | **Weed growth stage at measurement** | **Factor investigated** | **Factor value** | **Metric** | **Water use** |
| --- | --- | --- | --- | --- | --- | --- | --- | --- |
| buffalo bur  (*Solanum rostratum* Dunal) | UR | A(G) | - | Varies depending on year investigated | No external factor | NA | WUE^-1^ | 536 ml H_2_O g^-1^ d. wt. |
| Canada thistle (*Cirsium arvense* (L.) Scop.) | B8, G9 | T(F) | corn | 63-65 BBCH‡ | No external factor | NA | T_q_ | 0.016 kg H_2_O d^-1^ |
|  |  | S(F) | vineyard | 16/19 BBCH; 1.22 LAI |  |  | T | 2.1 mm H_2_O d^-1^ m^-2^ of soil |
| common cocklebur  (*Xanthium strumarium* L.) | UR | D(F) | - | - | T (°C) | 35 | T | 3.2 g H_2_O dm^-2^ hr^-1^ |
|  |  |  | - | - |  | 28 | T | 1.9 g H_2_O dm^-2^ hr^-1^ |
|  |  | O(F) | soybean | 2640 cm^2^ leaf area | No external factor | NA | T_q_ | 653 g H_2_O plant^-1^ |
|  |  | A(G) | - | Varies depending on year investigated |  |  | WUE^-1^ | 415 ml H_2_O g^-1^ d. wt. |
| common lambsquarters  (*Chenopodium album* L.) | B3 | S(F) | vineyard | 16/18 BBCH; 0.76 LAI | No external factor | NA | T | 2.2 mm H_2_O d^-1^ m^-2^ of soil |
|  |  | B(G) | - | 0-45 DAP |  |  | WUE^-1^ | 435 ml H_2_O g^-1^ d. wt. |
|  |  | A(G) | - | Varies depending on year investigated |  |  | WUE^-1^ | 658 ml H_2_O g^-1^ d. wt. |
| common mallow  (*Malva neglecta* Wallr.) | UR | S(F) | vineyard | 51/55 BBCH; 0.93 LAI | No external factor | NA | T | 4.5 mm H_2_O d^-1^ m^-2^ of soil |
| common mugwort  (*Artemisia vulgaris* L.) | UR | T(F) | - | 67-73 BBCH | No external factor | NA [2006,  2007]^‡‡^ | T_q_ | 0.077 kg H_2_O d^-1^ |
|  |  |  | - | 67-75 BBCH |  |  | T_q_ | 0.084 kg H_2_O d^-1^ |
| common purslane  (*Portulaca oleracea* L.) | UR | B(G) | - | 0-45 DAP | No external factor | NA | WUE^-1^ | 288 ml H_2_O g^-1^ d. wt. |
|  |  | A(G) | - | Varies depending on year investigated |  |  | WUE^-1^ | 281 ml H_2_O g^-1^ d. wt. |
| common reed  (*Phragmites australis* (Cav.) Trin. ex Steud.) | UR | W(G) | - | 42 DAP; 18.1 cm^2^ leaf area | Haplotypes, T (°C), CO_2_ (ppm) | Eurasian haplotype, 32/31, 400 | T | 5.8 mmol H_2_O m^-2^ s^-1^ |
|  |  |  | - | 42 DAP; 18.5 cm^2^ leaf area |  | Eurasian haplotype, 35/24, 650 | T | 6.2 mmol H_2_O m^-2^ s^-1^ |
|  |  |  | - | 42 DAP; 44.3 cm^2^ leaf area |  | Gulf Coast haplotype, 32/31, 400 | T | 3.7 mmol H_2_O m^-2^ s^-1^ |
|  |  |  | - | 42 DAP; 38.3 cm^2^ leaf area |  | Gulf Coast haplotype, 35/24; 650 | T | 6 mmol H_2_O m^-2^ s^-1^ |
| common sunflower  (*Helianthus annuus* L.) | UR | A(G) | - | Varies depending on year investigated | No external factor | NA | WUE^-1^ | 577 ml H_2_O g^-1^ d. wt. |
| dandelion  (*Taraxacum officinale* F.H. Wigg.) | UR | S(F) | vineyard | 15/18 BBCH; 1.38 LAI | No external factor |  | T | 2.5 mm H_2_O d^-1^ m^-2^ of soil |
| entire morningglory (*Ipomoea hederacea* var. *integriuscula* Gray) | UR | D(F) | - | - | T (°C) | 35 | T | 2.4 g H_2_O dm^-2^ hr^-1^ |
|  |  |  | - | - |  | 28 | T | 2.2 g H_2_O dm^-2^ hr^-1^ |
| horseweed (*Erigeron canadensis* L.) | B5, G4 | T(F) | - | 69-89 BBCH | No external factor | NA [2006,  2007] | T_q_ | 0.116 kg H_2_O d^-1^ |
|  |  |  | - |  |  |  | T_q_ | 0.174 kg H_2_O d^-1^ |
| ivyleaf morningglory (*Ipomoea hederacea* Jacq.) | B8, G9 | D(F) | - | - | T (°C) | 35 | T | 2.8 g H_2_O dm^-2^ hr^-1^ |
|  |  |  | - | - |  | 28 | T | 2.5 g H_2_O dm^-2^ hr^-1^ |
|  |  | N(G) | - | 22-29 DAP; 413 cm^2^ | Herbicide(s); crop-weed interaction | Nontreated; 1 IM plant | ET | 32 ml H_2_O d^-1^ |
|  |  |  | - | 22-29 DAP; 8 cm^2^ |  | Chlorimuron + metribuzin; 1 IM plant | ET | 2 ml H_2_O d^-1^ |
|  |  |  | - | 22-29 DAP; 10 cm^2^ |  | Imazaquin; 1 IM plant | ET | 2 ml H_2_O d^-1^ |
|  |  |  | - | 22-29 DAP; 304 cm^2^ |  | Nontreated; 2 IM plant | ET | 37 ml H_2_O d^-1^ |
|  |  |  | - | 22-29 DAP; 8 cm^2^ |  | Chlorimuron + metribuzin; 2 IM plant | ET | 1 ml H_2_O d^-1^ |
|  |  |  | - | 22-29 DAP; 19 cm^2^ |  | Imazaquin; 2 IM plant | ET | 4 ml H_2_O d^-1^ |
|  |  |  | Soybean | 22-29 DAP; 289 cm^2^ (IM§ )+ 299 cm^2^ (soy¶) |  | Nontreated; 1 IM plant + 1 soy plant | ET | 32 ml H_2_O d^-1^ |
|  |  |  |  | 22-29 DAP; 5 cm^2^ (IM) + 292 cm^2^ (soy) |  | Chlorimuron + metribuzin; 1 IM plant + 1 soy plant | ET | 23 ml H_2_O d^-1^ |
|  |  |  |  | 22-29 DAP; 15 cm^2^ (IM) + 291 cm^2^ (soy) |  | Imazaquin; 1 IM plant + 1 soy plant | ET | 20 ml H_2_O d^-1^ |
| jimsonweed (*Datura stramonium* L.) | UR | D(F) | - | - | T (°C) | 35 | T | 3 g H_2_O dm^-2^ hr^-1^ |
|  |  |  | - | - |  | 28 | T | 2.9 g H_2_O dm^-2^ hr^-1^ |
| johnsongrass (*Sorghum halepense* (L.) Pers.) | G9 | D(F) | - | - | T (°C) | 35 | T | 0.7 g H_2_O dm^-2^ hr^-1^ |
|  |  |  | - | - |  | 28 | T | 1.5 g H_2_O dm^-2^ hr^-1^ |
| jointed goatgrass (*Aegilops cylindrica* Host) | UR | I(G) | - | Advanced-tillered stage | Accession §§ | WA | T | 11.4 µg H_2_O cm^-2^ s^-1^ |
|  |  |  | - |  |  | OR | T | 10.1 µg H_2_O cm^-2^ s^-1^ |
|  |  |  | - |  |  | MT | T | 11.7 µg H_2_O cm^-2^ s^-1^ |
|  |  |  | - |  |  | WY | T | 10.9 µg H_2_O cm^-2^ s^-1^ |
|  |  |  | - |  |  | NE | T | 7.7 µg H_2_O cm^-2^ s^-1^ |
|  |  |  | - |  |  | CO | T | 10.8 µg H_2_O cm^-2^ s^-1^ |
|  |  |  | - |  |  | KS | T | 6.7 µg H_2_O cm^-2^ s^-1^ |
|  |  |  | - |  |  | KS | T | 7.8 µg H_2_O cm^-2^ s^-1^ |
|  |  |  | - |  |  | OK | T | 10.2 µg H_2_O cm^-2^ s^-1^ |
|  |  |  | - |  |  | WA | r_l_ | 1.8 s cm^-1^ |
|  |  |  | - |  |  | OR | r_l_ | 2.2 s cm^-1^ |
|  |  |  | - |  |  | MT | r_l_ | 1.8 s cm^-1^ |
|  |  |  | - |  |  | WY | r_l_ | 2 s cm^-1^ |
|  |  |  | - |  |  | NE | r_l_ | 3.3 s cm^-1^ |
|  |  |  | - |  |  | CO | r_l_ | 1.9 s cm^-1^ |
|  |  |  | - |  |  | KS | r_l_ | 3.9 s cm^-1^ |
|  |  |  | - |  |  | KS | r_l_ | 3.2 s cm^-1^ |
|  |  |  | - |  |  | OK | r_l_ | 2.1 s cm^-1^ |
|  |  | H(F) | - | 14 DAP | No external factor | NA | T | 51.2 µg H_2_O s^-1^ g^-1^ d. wt. of spike |
|  |  |  | - |  |  |  | r_l_ | 2.3 s cm^-1^ |
|  |  | J(G) | - | Five tillers | PPFD (uE m^-2^ s^-1^) | 125 | T | 4.7 ml H_2_O d^-1^ |
|  |  |  | - |  |  | 250 | T | 6 µg H_2_O cm^-2^ s^-1^ |
|  |  |  | - |  |  | 400 | T | 6.8 µg H_2_O cm^-2^ s^-1^ |
|  |  |  | - |  |  | 800 | T | 7.6 µg H_2_O cm^-2^ s^-1^ |
|  |  |  | - |  |  | 1100 | T | 7.9 µg H_2_O cm^-2^ s^-1^ |
|  |  |  | - |  |  | 1400 | T | 8 µg H_2_O cm^-2^ s^-1^ |
|  |  |  | - |  |  | 1850 | T | 8.4 µg H_2_O cm^-2^ s^-1^ |
|  |  |  | - |  | Leaf T (°C) | 10 | T | 2 µg H_2_O cm^-2^ s^-1^ |
|  |  |  | - |  |  | 15 | T | 5.2 µg H_2_O cm^-2^ s^-1^ |
|  |  |  | - |  |  | 20 | T | 7.1 µg H_2_O cm^-2^ s^-1^ |
|  |  |  | - |  |  | 25 | T | 8.9 µg H_2_O cm^-2^ s^-1^ |
|  |  |  | - |  |  | 30 | T | 10 µg H_2_O cm^-2^ s^-1^ |
|  |  |  | - |  |  | 35 | T | 10.8 µg H_2_O cm^-2^ s^-1^ |
|  |  |  | - |  |  | 40 | T | 13 µg H_2_O cm^-2^ s^-1^ |
| mayweed chamomile (*Anthemis cotula* L.) | UR | M(G) | - | 5-15 cm^2^ individual leaf area | PPFD (uE m^-2^ s^-1^); herbicide | 50; untreated | T | 3.8 µg H_2_O cm^-2^ s^-1^ |
|  |  |  | - |  |  | 100; untreated | T | 4 µg H_2_O cm^-2^ s^-1^ |
|  |  |  | - |  |  | 200; untreated | T | 4.6 µg H_2_O cm^-2^ s^-1^ |
|  |  |  | - |  |  | 400; untreated | T | 5.1 µg H_2_O cm^-2^ s^-1^ |
|  |  |  | - |  |  | 800; untreated | T | 5.9 µg H_2_O cm^-2^ s^-1^ |
|  |  |  | - |  |  | 1200; untreated | T | 6.4 µg H_2_O cm^-2^ s^-1^ |
|  |  |  | - |  |  | 1800; untreated | T | 6.7 µg H_2_O cm^-2^ s^-1^ |
|  |  |  | - |  |  | 85; metribuzin-treated | T | 3.6 µg H_2_O cm^-2^ s^-1^ |
|  |  |  | - |  |  | 335; metribuzin-treated | T | 3.6 µg H_2_O cm^-2^ s^-1^ |
|  |  |  | - |  |  | 1850; metribuzin-treated | T | 4.1 µg H_2_O cm^-2^ s^-1^ |
| mountain sage (*Artemisia frigida*) | UR | A(G) | - | Varies depending on year investigated | No external factor | NA | WUE^-1^ | 654 ml H_2_O g^-1^ d.wt. |
| nightshade (*Solanum* spp.) | UR |  | - |  | No external factor |  | WUE^-1^ | 587 ml H_2_O g^-1^ d.wt. |
| Palmer amaranth (*Amaranthus palmeri* S. Watson) | B1, G1 | Q(F) | corn | 30 DAP until corn physiological maturity | Weed density (plants m^-1^) | 0 | ET | 67.4 cm H_2_O |
|  |  |  |  |  |  | 0.5 | ET | 68.9 cm H_2_O |
|  |  |  |  |  |  | 1 | ET | 69.6 cm H_2_O |
|  |  |  |  |  |  | 2 | ET | 70.4 cm H_2_O |
|  |  |  |  |  |  | 4 | ET | 71 cm H_2_O |
|  |  |  |  |  |  | 8 | ET | 71.5 cm H_2_O |
|  |  | U(F) | cotton | 57 DAP-123 DAP | No external factor | NA | T | 1.2 g H_2_O cm^-2^ d^-1^ |
|  |  |  |  |  |  |  | g_l_ | 0.4 mol H_2_O m^-2^ s^-1^ |
|  |  | D(F) | - | - | T (°C) | 35 | T | 3.9 g H_2_O dm^-2^ hr^-1^ |
|  |  |  | - | - |  | 28 | T | 1.7 g H_2_O dm^-2^ hr^-1^ |
| perennial ryegrass (*Lolium perenne* L.) | UR | P(G) | - | 66-103 DAP | Water deficit (MPa) | 0 | T | 1024 ml H_2_O plant^-1^ |
|  |  |  | - |  |  | -0.5 | T | 569 ml H_2_O plant^-1^ |
|  |  |  | - |  |  | -1 | T | 400 ml H_2_O plant^-1^ |
| perennial sowthistle (*Sonchus arvensis* L.) | UR | K(G) | - | 42 DAP; 320 dm^2^ plant^-1^ | Soil water (bar) | 0 | T | 5.5 mmol H_2_O m^-2^ s^-1^ |
|  |  |  | - | 42 DAP; 198 dm^2^ plant^-1^ |  | -0.33 | T | 4.5 mmol H_2_O m^-2^ s^-1^ |
|  |  |  | - | 42 DAP; 59 dm^2^ plant^-1^ |  | -1 | T | 3.1 mmol H_2_O m^-2^ s^-1^ |
|  |  |  | - | 42 DAP; 37 dm^2^ plant^-1^ |  | -2 | T | 2 mmol H_2_O m^-2^ s^-1^ |
|  |  |  | - | 42 DAP; 18 dm^2^ plant^-1^ |  | -5 | T | 1.7 mmol H_2_O m^-2^ s^-1^ |
|  |  |  | - | 42 DAP; 184 dm^2^ plant^-1^ | PPFD (µE m^-2^ s^-1^) | 1015 | T | 4.9 mmol H_2_O m^-2^ s^-1^ |
|  |  |  | - | 42 DAP; 289 dm^2^ plant^-1^ |  | 580 | T | 3.7 mmol H_2_O m^-2^ s^-1^ |
|  |  |  | - | 42 DAP; 187 dm^2^ plant^-1^ |  | 285 | T | 1.5 mmol H_2_O m^-2^ s^-1^ |
|  |  |  | - | 42 DAP; 86 dm^2^ plant^-1^ | T (°C) | 30/25 | T | 9.7 mmol H_2_O m^-2^ s^-1^ |
|  |  |  | - | 42 DAP; 227 dm^2^ plant^-1^ |  | 20/15 | T | 4 mmol H_2_O m^-2^ s^-1^ |
|  |  |  | - | 42 DAP; 45 dm^2^ plant^-1^ |  | 10/5 | T | 0.6 mmol H_2_O m^-2^ s^-1^ |
|  |  |  | - | 42 DAP; 320 dm^2^ plant^-1^ | Soil water (bar) | 0 | g_l_ | 231 mmol H_2_O m^-2^ s^-1^ |
|  |  |  | - | 42 DAP; 198 dm^2^ plant^-1^ |  | -0.33 | g_l_ | 157 mmol H_2_O m^-2^ s^-1^ |
|  |  |  | - | 42 DAP; 59 dm^2^ plant^-1^ |  | -1 | g_l_ | 100 mmol H_2_O m^-2^ s^-1^ |
|  |  |  | - | 42 DAP; 37 dm^2^ plant^-1^ |  | -2 | g_l_ | 60 mmol H_2_O m^-2^ s^-1^ |
|  |  |  | - | 42 DAP; 18 dm^2^ plant^-1^ |  | -5 | g_l_ | 53 mmol H_2_O m^-2^ s^-1^ |
|  |  |  | - | 42 DAP; 184 dm^2^ plant^-1^ | PPFD (µE m^-2^ s^-1^) | 1015 | g_l_ | 192 mmol H_2_O m^-2^ s^-1^ |
|  |  |  | - | 42 DAP; 289 dm^2^ plant^-1^ |  | 580 | g_l_ | 137 mmol H_2_O m^-2^ s^-1^ |
|  |  |  | - | 42 DAP; 187 dm^2^ plant^-1^ |  | 285 | g_l_ | 67 mmol H_2_O m^-2^ s^-1^ |
|  |  |  | - | 42 DAP; 86 dm^2^ plant^-1^ | T (°C) | 30/25 | g_l_ | 580 mmol H_2_O m^-2^ s^-1^ |
|  |  |  | - | 42 DAP; 227 dm^2^ plant^-1^ |  | 20/15 | g_l_ | 343 mmol H_2_O m^-2^ s^-1^ |
|  |  |  | - | 42 DAP; 45 dm^2^ plant^-1^ |  | 10/5 | g_l_ | 57 mmol H_2_O m^-2^ s^-1^ |
| prickly lettuce (*Lactuca serriola* L.) | UR | T(F) | - | 67-81 BBCH | No external factor | NA [2006,  2007] | T_q_ | 0.153 kg H_2_O d^-1^ |
|  |  |  | - | 63-85 BBCH |  |  | T_q_ | 0.093 kg H_2_O d^-1^ |
| prickly pear (*Opuntia polyacantha*) | UR | C(G) | - | 9-month old | Light/Dark (hours), RH (%) | 7 dark, 47 | T | 0.06 g H_2_O hr^-1^ |
|  |  |  | - |  |  | 17 light, 47 | T | 0.06 g H_2_O hr^-1^ |
|  |  |  | - |  |  | 7 dark, 47 | T | 0.06 g H_2_O hr^-1^ |
|  |  |  | - |  |  | 17 light, 90 | T | 0.02 g H_2_O hr^-1^ |
|  |  |  | - |  |  | 7 dark, 80 | T | 0.01 g H_2_O hr^-1^ |
|  |  |  | - |  |  | 17 light, 54 | T | 0.12 g H_2_O hr^-1^ |
| prostrate knotweed (*Polygonum aviculare* L.) | UR | A(G) | - | Varies depending on year investigated | No external factor | NA | WUE^-1^ | 678 ml H_2_O g^-1^ d.wt. |
| quackgrass (*Elymus repens* (L.) Gould) | UR | K(F) | alfalfa | 35 cm height | Postemergence herbicide | Quizalofop | T | 2.3 µg H_2_O cm^-2^ s^-1^ |
|  |  |  |  |  |  | Haloxyfop | T | 1.3 µg H_2_O cm^-2^ s^-1^ |
|  |  |  |  |  |  | Fluazifop-P | T | 2.9 µg H_2_O cm^-2^ s^-1^ |
|  |  |  |  |  |  | Control | T | 7.2 µg H_2_O cm^-2^ s^-1^ |
|  |  |  |  |  |  | Quizalofop | T | 3.3 µg H_2_O cm^-2^ s^-1^ |
|  |  |  |  |  |  | Haloxyfop | T | 0.9 µg H_2_O cm^-2^ s^-1^ |
|  |  |  |  |  |  | Fluazifop-P | T | 4.3 µg H_2_O cm^-2^ s^-1^ |
|  |  |  |  |  |  | Control | T | 10.3 µg H_2_O cm^-2^ s^-1^ |
|  |  |  |  |  |  | Quizalofop | r_l_ | 21.7 s cm^-1^ |
|  |  |  |  |  |  | Haloxyfop | r_l_ | 19.7 s cm^-1^ |
|  |  |  |  |  |  | Fluazifop-P | r_l_ | 15.6 s cm^-1^ |
|  |  |  |  |  |  | Control | r_l_ | 2.3 s cm^-1^ |
|  |  |  |  |  |  | Quizalofop | r_l_ | 14.6 s cm^-1^ |
|  |  |  |  |  |  | Haloxyfop | r_l_ | 26.3 s cm^-1^ |
|  |  |  |  |  |  | Fluazifop-P | r_l_ | 6.7 s cm^-1^ |
|  |  |  |  |  |  | Control | r_l_ | 1.1 s cm^-1^ |
| ragweed parthenium (*Parthenium hysterophorus* L.) | UR | R(F) | - | 5.8 LAI | T (°C); CO_2_ (µmol CO_2_ m^-2^ s^-1^) | 25; 360 | T | 3.3 mmol H_2_O m^-2^ s^-1^ |
|  |  |  | - |  |  | 30; 360 | T | 9.7 mmol H_2_O m^-2^ s^-1^ |
|  |  |  | - |  |  | 30; 700 | T | 2.8 mmol H_2_O m^-2^ s^-1^ |
|  |  |  | - |  |  | 25; 700 | T | 2.5 mmol H_2_O m^-2^ s^-1^ |
|  |  |  | - |  |  | 30; 360 | g_l_ | 0.3 mmol H_2_O m^-2^ s^-1^ |
|  |  |  | - |  |  | 30; 700 | g_l_ | 0.2 mmol H_2_O m^-2^ s^-1^ |
|  |  |  | - |  |  | 25; 700 | g_l_ | 0.2 mmol H_2_O m^-2^ s^-1^ |
| redroot pigweed (*Amaranthus retroflexus* L.) | B7 | B(G) | - | 0-31 DAP, 0-44 DAP | No external factor | NA | WUE^-1^ | 261 ml H_2_O g^-1^ d.wt. |
|  |  | A(G) | - | Varies depending on year investigated |  |  | WUE^-1^ | 305 ml H_2_O g^-1^ d.wt. |
|  |  | T(F) | - | 69-75 BBCH |  |  | T_q_ | 0.018 kg H_2_O d^-1^ |
| Russian thistle (*Salsola tragus* L.) | UR | B(G) | - | 0-31 DAP, 0-43 DAP | No external factor |  | WUE^-1^ | 224 ml H_2_O g^-1^ d.wt. |
|  |  | A(G) | - | Varies depending on year investigated |  |  | WUE^-1^ | 314 ml H_2_O g^-1^ d.wt. |
| showy crotalaria (*Crotalaria spectabilis* Roth) | UR | E(G) | - | 35 DAP; 16 dm^2^ | CO_2_ (ppm); Nutrient strength | 350; 1/8 | T | 5.2 g H_2_O dm^-2^ hr^-1^ |
|  |  |  | - | 35 DAP; 26 dm^2^ |  | 350; 1/2 | T | 5.3 g H_2_O dm^-2^ hr^-1^ |
|  |  |  | - | 35 DAP; 17 dm^2^ |  | 675; 1/8 | T | 3.2 g H_2_O dm^-2^ hr^-1^ |
|  |  |  | - | 35 DAP; 40 dm^2^ |  | 675; 1/2 | T | 3.6 g H_2_O dm^-2^ hr^-1^ |
|  |  |  | - | 35 DAP; 16 dm^2^ |  | 350; 1/8 | r_l_ | 0.4 s cm^-1^ |
|  |  |  | - | 35 DAP; 26 dm^2^ |  | 350; 1/2 | r_l_ | 0.4 s cm^-1^ |
|  |  |  | - | 35 DAP; 17 dm^2^ |  | 675; 1/8 | r_l_ | 0.7 s cm^-1^ |
|  |  |  | - | 35 DAP; 40 dm^2^ |  | 675; 1/2 | r_l_ | 0.6 s cm^-1^ |
| sicklepod (*Senna obtusifoli*a (L.) H.S. Irwin & Barneby) | UR | O(F) | soybean | 1640 cm^2^ leaf area | No external factor | NA | T_q_ | 230 g H_2_O plant^-1^ |
|  |  | E(G) | - | 35 DAP; 13 dm^2^ | CO_2_ (ppm); Nutrient strength | 350; 1/8 | T | 4.6 g H_2_O dm^-2^ hr^-1^ |
|  |  |  | - | 35 DAP; 44 dm^2^ |  | 350; 1/2 | T | 5.5 g H_2_O dm^-2^ hr^-1^ |
|  |  |  | - | 35 DAP; 15 dm^2^ |  | 675; 1/8 | T | 3.0 g H_2_O dm^-2^ hr^-1^ |
|  |  |  | - | 35 DAP; 59 dm^2^ |  | 675; 1/2 | T | 3.5 g H_2_O dm^-2^ hr^-1^ |
|  |  |  | - | 35 DAP; 13 dm^2^ |  | 350; 1/8 | r_l_ | 0.4 s cm^-1^ |
|  |  |  | - | 35 DAP; 44 dm^2^ |  | 350; 1/2 | r_l_ | 0.3 s cm^-1^ |
|  |  |  | - | 35 DAP; 15 dm^2^ |  | 675; 1/8 | r_l_ | 0.8 s cm^-1^ |
|  |  |  | - | 35 DAP; 59 dm^2^ |  | 675; 1/2 | r_l_ | 0.6 s cm^-1^ |
| smooth brome (*Bromus inermis* Leyss.) | G3 | B(G) | - | 0-121 DAP, 0-93 DAP, 0-93 DAP, 0-112 DAP | No external factor | NA | WUE^-1^ | 784 ml H_2_O g^-1^ d.wt. |
|  |  | A(G) | - | Varies depending on year investigated |  |  | WUE^-1^ | 977 ml H_2_O g^-1^ d.wt. |
| smooth pigweed (*Amaranthus hybridus* L.) | B7 | F(F) | cotton | July 17, 1981 | Soil water status**; Leaf side | High; Abaxial | T | 22.5 µg H_2_O cm^-2^ s^-1^ |
|  |  |  |  |  |  | Low; Abaxial | T | 18.3 µg H_2_O cm^-2^ s^-1^ |
|  |  |  |  | Aug 29, 1981 |  | High; Abaxial | T | 9.2 µg H_2_O cm^-2^ s^-1^ |
|  |  |  |  |  |  | High; Adaxial | T | 11.0 µg H_2_O cm^-2^ s^-1^ |
|  |  |  |  |  |  | High; Abaxial | T | 9.2 µg H_2_O cm^-2^ s^-1^ |
|  |  |  |  |  |  | High; Adaxial | T | 8.0 µg H_2_O cm^-2^ s^-1^ |
| velvetleaf (*Abutilon theophrasti* Medik.) | UR | G(F) | soybean | Anthesis | Monocultured/intercropped soybean/weed (plants m^-2^); measurement time (hr) | Monocultured soybean (32.5); 1000 | T | 165.4 mg H_2_O m^-2^ s^-1^ |
|  |  |  |  |  |  | Intercropped soybean (32.5, 5); 1000 | T | 218.1 mg H_2_O m^-2^ s^-1^ |
|  |  |  |  |  |  | Monocultured weed (5); 1000 | T | 218.9 mg H_2_O m^-2^ s^-1^ |
|  |  |  |  |  |  | Intercropped weed (5, 32.5); 1000 | T | 193.8 mg H_2_O m^-2^ s^-1^ |
|  |  |  |  |  |  | Monocultured soybean (32.5); 1400 | T | 266.7 mg H_2_O m^-2^ s^-1^ |
|  |  |  |  |  |  | Intercropped soybean (32.5, 5); 1400 | T | 189.4 mg H_2_O m^-2^ s^-1^ |
|  |  |  |  |  |  | Monocultured weed (5); 1400 | T | 267.2 mg H_2_O m^-2^ s^-1^ |
|  |  |  |  |  |  | Intercropped weed (5, 32.5); 1400 | T | 141.9 mg H_2_O m^-2^ s^-1^ |
|  |  |  |  |  |  | Monocultured soybean (32.5); 1000 | g_l_ | 1.0 cm s^-1^ |
|  |  |  |  |  |  | Intercropped soybean (32.5, 5) ; 1000 | g_l_ | 1.0 cm s^-1^ |
|  |  |  |  |  |  | Monocultured weed (5); 1000 | g_l_ | 1.5 cm s^-1^ |
|  |  |  |  |  |  | Intercropped weed (5, 32.5); 1000 | g_l_ | 0.8 cm s^-1^ |
|  |  |  |  |  |  | Monocultured soybean (32.5); 1400 | g_l_ | 1.1 cm s^-1^ |
|  |  |  |  |  |  | Intercropped soybean (32.5, 5); 1400 | g_l_ | 0.6 cm s^-1^ |
|  |  |  |  |  |  | Monocultured weed (5); 1400 | g_l_ | 1.1 cm s^-1^ |
|  |  |  |  |  |  | Intercropped weed (5, 32.5); 1400 | g_l_ | 0.4 cm s^-1^ |
|  |  | V(G) | - | 41 DAP; 258 cm^2^ plant^-1^ | Water Availability†† | Low [2007] | T | 0.30 kg H_2_O plant^-1^ |
|  |  |  | - | 41 DAP; 329 cm^2^ plant^-1^ |  | Medium [2007] | T | 0.44 kg H_2_O plant^-1^ |
|  |  |  | - | 41 DAP; 688 cm^2^ plant^-1^ |  | High [2007] | T | 0.84 kg H_2_O plant^-1^ |
|  |  |  | - | 61 DAP; 961 cm^2^ plant^-1^ |  | Low [2007] | T | 1.81 kg H_2_O plant^-1^ |
|  |  |  | - | 61 DAP; 1293 cm^2^ plant^-1^ |  | Medium [2007] | T | 2.49 kg H_2_O plant^-1^ |
|  |  |  | - | 61 DAP; 2964 cm^2^ plant^-1^ |  | High [2007] | T | 6.37 kg H_2_O plant^-1^ |
|  |  |  | - | 77 DAP; 1046 cm^2^ plant^-1^ |  | Low [2007] | T | 8.16 kg H_2_O plant^-1^ |
|  |  |  | - | 77 DAP; 2122 cm^2^ plant^-1^ |  | Medium [2007] | T | 11.05 kg H_2_O plant^-1^ |
|  |  |  | - | 77 DAP; 5374 cm^2^ plant^-1^ |  | High [2007] | T | 19.72 kg H_2_O plant^-1^ |
|  |  |  | - | 42 DAP; 13 cm^2^ plant^-1^ |  | Low [2008] | T | 0.19 kg H_2_O plant^-1^ |
|  |  |  | - | 42 DAP; 26 cm^2^ plant^-1^ |  | Medium [2008] | T | 0.21 kg H_2_O plant^-1^ |
|  |  |  | - | 42 DAP; 103 cm^2^ plant^-1^ |  | High [2008] | T | 0.38 kg H_2_O plant^-1^ |
|  |  |  | - | 61 DAP; 33 cm^2^ plant^-1^ |  | Low [2008] | T | 0.27 kg H_2_O plant^-1^ |
|  |  |  | - | 61 DAP; 792 cm^2^ plant^-1^ |  | Medium [2008] | T | 1.65 kg H_2_O plant^-1^ |
|  |  |  | - | 61 DAP; 1209 cm^2^ plant^-1^ |  | High [2008] | T | 4.19 kg H_2_O plant^-1^ |
|  |  |  | - | 75 DAP; 407 cm^2^ plant^-1^ |  | Low [2008] | T | 1.38 kg H_2_O plant^-1^ |
|  |  |  | - | 75 DAP; 1145 cm^2^ plant^-1^ |  | Medium [2008] | T | 5.77 kg H_2_O plant^-1^ |
|  |  |  | - | 75 DAP; 2208 cm^2^ plant^-1^ |  | High [2008] | T | 9.92 kg H_2_O plant^-1^ |
|  |  | G(G) | - | Flowering | Leaf water potential (MPa) | -1.5 | T | 278.7 mg H_2_O m^-2^ s^-1^ |
|  |  |  | - |  |  | -1.9 | T | 151 mg H_2_O m^-2^ s^-1^ |
|  |  |  | - |  |  | -2.3 | T | 72.6 mg H_2_O m^-2^ s^-1^ |
|  |  |  | - |  |  | -2.7 | T | 43.4 mg H_2_O m^-2^ s^-1^ |
| witchgrass (*Panicum capillare* L.) | UR | B(G) | - | 0-46 DAP, 0-44 DAP | No external factor | NA | WUE^-1^ | 254 ml H_2_O g^-1^ d.wt. |

* Ranking assigned to weed species; B: Top 10 troublesome weeds (B1-B10) among all broadleaf crops, fruits and vegetables as per 2019 WSSA National weed survey dataset, G: Top 10 troublesome weeds (G1-G10) among all grass crops, pasture and turf as per 2020 WSSA National weed survey dataset, where UR means “unranked” and is designated to weed species exclusive of these datasets. † Alphabetic code given to publications along with “F” or “G” in the parenthesis indicating whether study was conducted in “Field” or “Greenhouse”. ‡ BBCH: Biologische Bundesanstalt, Bundessortenamt und CHemische Industrie scale used to describe the phenological development stage of different plant species. The BBCH scale has two digits; the first and second digit represent principal and secondary growth stages, respectively. § IM: Ivyleaf morningglory. ¶ Soy: soybean. ** Soil water status; High: Preplant and subsequent irrigation of 10 cm in late June. Low: Preplant irrigation only. †† Water availability; Low: Fraction of transpirable soil water (FTSW) at which one-third of transpiration occurred. Medium: FTSW at which two-thirds of transpiration occurred. High: FTSW at which full transpiration occurred. ‡‡ If WU data is given separately for more than one-year, specific year corresponding to the respective WU values is given in the square bracket in the “Factor value” column. §§ Abbreviated values of “Accession” factor represent the states of the USA.
